# Supplementary material for: Defects induce phase transition from dynamic to static rippling in graphene
Source: Proc Natl Acad Sci U S A. 2025 Feb 28;122(9):e2416932122. doi: 10.1073/pnas.2416932122 (PMC11892612; doi:10.1073/pnas.2416932122)
Supplement: Supplementary file 1 — Appendix 01 (PDF) [file pnas.2416932122.sapp.pdf]

Supporting Information for

Defects induce phase transition from dynamic to static rippling in graphene

Fabian L. Thiemann, Camille Scalliet, Erich A. Müller, Angelos Michaelides

Fabian L Thiemann, Camille Scalliet, and Angelos Michaelides.
E-mail: fabian.thiemann@ibm.com, camille.scalliet@ens.fr, am452@cam.ac.uk

This PDF file includes:

Supporting text
Figs. S1 to S24
Table S1
Legends for Movies S1 to S5
SI References

Other supporting materials for this manuscript include the following:

Movies S1 to S5

Supporting Information Text

In this supplementary information, we provide additional details on certain aspects of the study reported in the manuscript. This includes a detailed summary of the computational details and methods as well as an analysis of the sensitivity of the results.

S1. Computational details

S1.A. Molecular dynamics simulation. For all our classical molecular dynamics (MD) simulations, we employed monoclinic simulation cells where the length of the in-plane lattice vectors was ≈ 14.6 nm. Periodic boundary conditions were applied in all three directions. We ensured the graphene sheet did not interact with its periodic images perpendicular to the surface by adding a 5 nm vacuum, significantly surpassing the cutoff of the GAP-20 (1 nm) (1). The number of atoms varied with the type and number of defects introduced. While the pristine graphene sheet comprised 7200 atoms, systems of the highest divacancy defect concentration investigated (1.0% corresponding to 36 divacancy defects) contained 7128 atoms. Graphene sheets with Stone-Wales defects, conversely, were of identical size as their pristine reference, as this defect is formed by bond rotation rather than atom removal. For all defective systems, the defects were distributed randomly with random orientations across the sheet while satisfying a minimum distance criterion of 1 nm between the defect centers. To sample the impact of different realisations of a respective defect concentration in the transition region, for divacancy defects we went beyond the suggested 3 samples (2) and simulated 5 different spatial arrangements for systems comprising between 3 and 8 defects corresponding to defect concentrations between $\approx 0.083\%$ and $\approx 0.22\%$. For the systems with 2, 12, 20, and 36 divacancies, and sheets with 2, 3, 4, 8, and 36 Stone-Wales defects, two realisations of the respective defect density were generated. For membranes comprising only 1 divacancy and 20 Stone-Wales defects, respectively, we only performed one simulation for a specific arrangement. This was considered sufficient due to the small differences in the properties of interest between independent systems of these defect densities.

All simulations were performed with a timestep of 1 fs at a temperature of 300 K and zero strain in the isobaric-isothermal ensemble (NPT). The target temperature and stress were ensured by employing a Nosé-Hoover chain thermostat and barostat where the barostat was only applied to the in-plane dimensions. These were coupled to each other to guarantee that graphene retains its hexagonal nature. The interaction between carbon atoms was described using the GAP-20 (1) which has been benchmarked in previous work (2) for graphene with a high density of divacancies achieving a maximum root mean square (rms) force error of ≈ 112.5 meV/Å with respect to its *ab initio* reference for defect concentrations of 3.0%. However, for the concentrations investigated in this work, a force error of ≈ 74.4 meV/Å was found. A thorough benchmarking of the potential with respect to the phonon dispersion curves and defect formation energies can be found in the original reference (1). All simulations were started from a perfectly flat sheet ($z = 0$ for all atoms) and each system was equilibrated for 50 ps. Statistics were then gathered over a minimum of 700 ps, however, production runs for most systems were at least of the length of 1 ns and up to 1.6 ns. In section S3.A.3, we show that the properties reported in the manuscript are in fact well converged for these simulation times. The atomic positions were saved every 100 fs. All simulations reported in the manuscript were performed using LAMMPS (3).

S1.B. Computation of observables.

S1.B.1. Atomic normal vector. The atomic normal vector is the central quantity of this work and provides valuable information about the corrugation and dynamics of the system. Here we provide a comprehensive overview of the definition and computation of this measure. The atomic normal vector, denoted as $\vec{n}_i$, represents the vector perpendicular to the local surface, A_i, of the graphene sheet, anchored at atom i. A schematic illustration of these quantities is shown in figure S2. To compute $\vec{n}_i$, we approximate the local surface, A_i, following an identical approach as in previous work (2). Specifically, we identify all atoms within a distance of 4.5 Å from atom i and express the coordinates of these neighboring atoms relative to atom i. The value 4.5 Å corresponds to the cutoff of the many-body SOAP (smooth overlap of atomic positions) descriptor used by GAP-20 (1). While each atom's local environment in pristine graphene comprised 22 atoms, this number was slightly reduced for atoms in proximity to divacancies and Stone-Wales defects in defective systems. We then fit a two-dimensional second-order polynomial

$$z(x, y) = a + bx + cy + dxy + ex^2 + fy^2 \ , \quad [1]$$

to the relative heights of these atoms to determine the local surface. For all atoms and frames in all systems the R^2 score of the fit exceeded 0.9 indicating an accurate description of the local surface A_i. We obtain the atomic normal vector $\vec{n}_i$ by taking the gradient of the surface equation 1 at (0,0,0), i.e. at atom i,

$$\vec{n}_i = [-b, -c, 1]^T \ . \quad [2]$$

S1.B.2. Atomic normal angle. The atomic normal angle, θ_i, is defined as the angle between the atomic normal vector $\vec{n}_i$, as defined in equation 2, and the unit vector in z-direction $\vec{e}_z = [0, 0, 1]^T$,

$$\cos\theta_i = \frac{\vec{n}_i \cdot \vec{e}_z}{||\vec{n}_i|| ||\vec{e}_z||}, \quad [3]$$

where the numerator corresponds to the dot product of the two vectors while the denominator is the product of their norms. Equation 3 further simplifies to

$$\theta_i = \arccos\left(\frac{1}{\sqrt{b^2+c^2+1}}\right), \qquad [4]$$

and only depends on the coefficients b and c of the local surface approximation function. The rms normal angle, which we refer to as rms inclination in the manuscript in line with reference (4), of the system can then be computed from the ensemble average

$$\theta_{\mathrm{rms}} = \sqrt{\langle\theta^2\rangle} = \left(\frac{1}{N_a N_f}\sum_{i=1}^{N_a}\sum_{j=1}^{N_f}\theta_i^2(t_j)\right)^{\frac{1}{2}}, \qquad [5]$$

where N_a and N_f are the number of atoms and frames, respectively, and $\theta_i(t_j)$ is the atomic normal angle at atom i measured at frame j, or equivalently time t_j. To quantify the uncertainty associated with our measured value θ_{rms} for a given system, we calculate the standard deviation based on bootstrapping. This involves dividing each trajectory into four blocks and performing 10,000 resampling iterations. The error bars presented in the manuscript are, unless stated otherwise, derived from a threefold standard deviation obtained via this method.

S1.B.3. Angle autocorrelation function. To explore the impact of defects on the dynamics of atomic fluctuations in graphene, we extend the analysis to include time-dependent information on the atomic normal angle defined in equation 4. Specifically, we focus on the angle autocorrelation function, $\Phi_\theta(\tau)$, defined as

$$\Phi_\theta(\tau) = \langle\theta(\tau)\theta(0)\rangle = \frac{1}{N_a N_f}\sum_{i}^{N_a}\sum_{j}^{N_f}(\theta_i(t_j+\tau)\theta_i(t_j)), \qquad [6]$$

where $\theta_i(t_j)$ is the atomic normal angle at atom i measured at time t_j, τ is the time lag, and the brackets $\langle ... \rangle$ correspond to an ensemble average. The angle autocorrelation function is related to the rms inclination via $\Phi_\theta(\tau=0) = \langle\theta^2\rangle = \theta^2_{\mathrm{rms}}$. We normalise the angle autocorrelation by the mean squared atomic angle to compare across different defect concentrations, defining the normalized angle autocorrelation function, $\mathcal{C}_\theta(\tau)$, as

$$\mathcal{C}_\theta(\tau) = \frac{\Phi_\theta(\tau)}{\Phi_\theta(0)} = \frac{\langle\theta(\tau)\theta(0)\rangle}{\langle\theta(0)\theta(0)\rangle} = \frac{\langle\theta(\tau)\theta(0)\rangle}{\langle\theta^2\rangle}. \qquad [7]$$

We compute it for time lags up to 5 ps which ensures the convergence of $\mathcal{C}_\theta(\tau)$ to a plateau value as shown in figure S3 for pristine graphene. We provide a comprehensive overview of the results for all systems in section S2.A. To accurately determine the plateau value of $\mathcal{C}_\theta(\tau)$, we fit it to an exponential decay

$$f(\tau) = A\exp(-k\tau) + c \qquad [8]$$

and analyse its behaviour as τ approaches infinity ($f(\tau\to\infty) = c$). Across all systems discussed in the manuscript, we achieve R^2 scores above 0.92. The standard deviation for $\mathcal{C}_\theta(\tau)$ at any τ, as well as the estimate for plateau value $f(\tau\to\infty)$, was computed using the same bootstrapping approach described for the rms normal angle. While the ensemble average of the angle autocorrelation function in equation 6 provides the global perspective, computing $\mathcal{C}_\theta(\tau)$ separately for each individual atom allows to gain mechanistic insights, as demonstrated in Figure 3 of the manuscript. However, these atomwise calculations inherently rely on less converged statistics. Consequently, relatively low R^2 scores are obtained for some atoms in certain systems, leading to relatively high standard deviations. A full overview of these metrics is given in section S2.B.

S2. Comprehensive overview of results

In this section, we present a comprehensive summary of all results and the corresponding estimated uncertainties discussed in the manuscript.

S2.A. Angle autocorrelation function. Here, we report the angle autocorrelation function along with their plateau value $\mathcal{C}_\theta(\tau\to\infty)$ for all systems investigated. The pristine reference case is illustrated above in figure S3, while defective systems with fewer than 8 divacancy defects (corresponding to a concentration of $\approx 0.22\%$) are shown in figure S4. Conversely, angle autocorrelation functions for higher divacancy concentrations are depicted in figure S5. The results for all systems comprising Stone-Wales defects are depicted in figure S6. For the sake of clarity, rather than plotting the fitted exponential function for each system, we merely highlight the plateau value estimated by the constant c in each plot.

S2.B. Atomic heat maps based on plateau value of angle autocorrelation function.

Shifting our focus from a global perspective to a local examination, we now transition from the ensemble average of the autocorrelation function to the atomwise calculation of the plateau value $\mathcal{C}_\theta(\tau)$. To assess the impact of less converged statistics on the results, we present accuracy metrics in table S1 for the fit of the exponential function to the atomic autocorrelation functions. Here, we restrict this analysis on systems comprising divacancy defects and similar convergence trends were found for graphene sheets with Stone-Wales defects. Specifically, for each system, we provide the lowest R^2 value across all atoms and the count of atoms with an R^2 lower than a threshold of 0.8. In general, we observe better convergence in systems with lower defect concentrations, where the R^2 scores exhibit a monotonically decreasing trend with the number of defects. For systems with four or fewer divacancy defects, equivalent to a concentration of approximately 0.111 %, we obtain R^2 scores above the 0.8 threshold for all atoms. In more defective sheets, conversely, we find a substantial number of atoms with R^2 below 0.8. Still, we note that for most systems less than 1 % of particles fail to meet the convergence criterion. Only in systems with 20 defects or more (concentrations $\geq 0.556\%$) do we observe up to ≈ 11 % of atoms below our R^2 threshold of 0.8. We believe this does not compromise our ability to make qualitative statements about the results and overall behavior of these highly defective systems.

We show the atom-resolved plateau values of the angle autocorrelation function, $\mathcal{C}_\theta(\tau \to \infty)$, and the associated errors for all systems investigated in this work in the figures S7 to S16. In line with the analysis of the R^2 scores, we note a rise in uncertainty with higher defect concentrations. Interestingly, we also find significant deviations in the uncertainty across different spatial realisations of a given defect density as for example for 5 or 8 divacancy defects shown in the figures S10 and S12, respectively. Despite these variations, errors remain minimal for the majority of atoms, allowing for qualitative insights into local dynamics.

S2.C. Atomic heat maps based on average absolute virial contribution in out-of-plane direction.

In figure S17, we present heat maps of the average absolute atomic virial contribution to the stress along the surface normal, $\langle |W_{zz}| \rangle$. These maps are computed for the selected systems containing Stone-Wales defects shown in figure (e-h) in manuscript. Similar to the graphene sheets with divacancies, we observe that these heat maps display patterns consistent with those seen in the heat maps derived from the plateau value of the angle autocorrelation function.

S3. Sensitivity of the obtained results

In this section, we assess the reliability of our results through convergence tests concerning the interatomic potential used, system size, and simulation time. Then, we compute and discuss alternative measures reported in the literature to quantify the static and dynamic behavior of ripples.

S3.A. Convergence tests.

S3.A.1. Interatomic potential and defect-defect interaction energy. We start our convergence analysis by examining the capability of the employed interatomic potential, the GAP-20 (1), to describe the systems and phenomena explored in this study. Given the interaction between defects emerges from the overlap of the induced strain fields and is then mediated through the membrane's elasticity, an accurate description of the phonon dispersion spectrum and elastic constants is of high importance. While the GAP-20 has indeed been shown to accurately reproduce these properties for graphene with respect to its *ab-initio* reference, here we aim to further validate its consistency with the DFT defect-defect interaction energy. Specifically, we focus on the interaction between two divacancies and define their interaction energy as

$$E^{\text{inter}} = E^{\text{total}} - 2 \cdot E^{\text{single}} + E^{\text{pristine}}, \qquad [9]$$

where E^{total} is the potential energy of a graphene sheet comprising two divacancies (within periodic boundary conditions), E^{single} corresponds to the energy of the equivalent system comprising only one divacancy, and E^{pristine} represents the energy of the equivalent pristine system.

The interaction energy strongly depends on the distance between the two defects as well as their orientation. For simplicity, we fix the orientation and focus on whether GAP-20 captures the features of the DFT interaction energy as the defect separation increases. We create 14×14 graphene supercells (392 atoms in the pristine state) with a box height of 25Å and introduce two divacancies with separations ranging from ≈ 6.52Å to ≈ 19.25Å. This supercell size ensures sufficient spacing between defects and limits periodic image interactions. We note here, however, that the GAP-20 has a cutoff of 4.5Å and 10Å for many-body and two-body interactions, respectively.

For each configuration, both the lattice vectors and atomic positions are relaxed using GAP-20. The individual terms in Eq. 9 are then evaluated for these relaxed configurations using both GAP-20 and DFT. The DFT calculations were performed following the same methodology used for generating the training data for GAP-20. Specifically, we utilized the Vienna Ab initio Simulation Package (VASP) (5–7), employing the nonlocal optB88-vdW functional (8). A plane-wave energy cutoff of 600 eV and Gaussian smearing with a width of 0.1 eV were used. Given the large system size, all calculations were restricted to the Gamma point for computational efficiency. The total energy was converged within the self-consistent cycle to 10^{-6} eV.

A summary of the results of our analysis is depicted in figure S18. On the right panel we report the defect interaction energy, E^{inter}, as a function of the defect separation distance computed with both the GAP-20 and DFT. Both methods predict a decrease in the absolute interaction energy with increasing separation. GAP-20 slightly overestimates the interaction but captures the qualitative trend very well. In particular, the GAP-20 accurately predicts the slightly lower absolute interaction energy for a defect separation of 14.99Å compared to 19.25Å. The right panel of figure S18 illustrates the configuration at a divacancy distance of 10.74Å after relaxation with the GAP-20. The defects induce a strong out-of-plane deformation of the graphene sheet. Interestingly, the local defect environments resemble the pringle and inclined configuration (parallel and antiparallel symmetries) reported previously (9, 10). Based on these findings, we are confident that the conclusions drawn in the manuscript are robust and qualitatively accurate.

S3.A.2. System size. It is well-established in the theory of flexible membranes (11–13) that the average amplitude of the ripples exhibits a power-law scaling with the dimensions of the system. Here, we explore how the measures introduced in the manuscript, namely the rms inclination, θ_{rms}, and the plateau value of the normalised angle autocorrelation function, $\mathcal{C}_\theta(\tau \to \infty)$, depend on the system size by performing additional MD simulations on systems where the pristine sheet contains 1800 atoms. This corresponds to a quarter of the system size used in the simulations to produce the results reported in the manuscript. Given their strong interactions and impact on the dynamics, we focus this analysis on divacancy defects, but expect similar system size dependence for graphene sheets with Stone-Wales defects. We employ the identical simulation setup as described in Section S1.A, where all simulations are at least 800 ps in length, with the majority exceeding one nanosecond. To achieve defect concentrations comparable to those reported in the manuscript we introduce up to 9 divacancy defects, corresponding to a defect concentration of 1%. For each concentration investigated, we conduct three simulations, varying the arrangement of defects according to the constraints outlined in Section S1.A. The only exceptions are the pristine graphene sheet and the lowest accessible concentration, where only one defect is introduced.

The summary of our analysis is presented in figure S19, with the rms inclination, θ_{rms}, shown on the left and the dynamic measure, $\mathcal{C}_\theta(\tau \to \infty)$, on the right, both as function of the defect concentration for both the original and the reduced system sizes. We note that the R^2 scores for fitting the exponential function in equation 8 to the normalized angle autocorrelation functions are expectedly slighty lower for smaller system sizes. Nevertheless, we still obtain reliable fits, with R^2 scores above 0.9 for the large majority of cases, and the minimum score above 0.75. Similar to the results reported in the manuscript, we observe a discontinuity in both measures for the smaller systems, with the discontinuity being more pronounced in the dynamics of the system. With decreasing system size, the phase transition shifts to larger defect concentrations of $\approx 0.2\%$, establishing our reported concentration in the manuscript of $\approx 0.1\%$ as the upper boundary for graphene sheets with sizes of the order of 1 μm, as utilised in experiments (14). This analysis raises concerns about direct comparisons between ab initio molecular dynamics (AIMD) studies and experimental observations on defective graphene. While the former may suggest that the membrane maintains liquid-like ripples even with the introduction of a single defect, experiments conducted on larger graphene sheets at the same defect density are expected to result in highly buckled structures.

Finally, we briefly discuss the implications of our analysis for pristine graphene. In fact, in the absence of defects, both measures appear to be independent of the system size. At first glance, this may seem to contradict the scaling relation observed in both MD simulations (15) and electron diffraction experiments (16), where the rms inclination, θ_{rms}, scales exponentially with the system size of the freestanding graphene sheet, L, according to $\theta_{\text{rms}} \propto \exp\left(-l/L\right)$. However, our results can be reconciled with these findings by understanding the parameter l as the inverse resolution of the measurement. While in TEM experiments (16) l corresponds to the electron coherence length, typically in the range of $5-20$ nm (14), the computational study (15) obtains the average angle of a small region of graphene via Gaussian interpolation, and l represents the width of the Gaussian. In this work, conversely, we do not perform any spatial averaging of the normal angle; instead, we compute the normal angle exclusively at the center of each atom, and the rms inclination of the system is based on the ensemble average of these atomic angles as defined in equation 5. Framing this in the approach used in (15), instead of using Gaussians, we work with Dirac functions corresponding to $l=0$. In line with the scaling relation, this results in the same estimate of θ_{rms} irrespective of the system size. Given the low ratios employed in TEM measurements, with commonly used electron coherence lengths of $5-20$ nm and graphene dimensions of ≈ 1 μm resulting in $l/L \leq 0.02$, this enables a direct comparison of our results with experiments.

S3.A.3. Simulation time. In this section we investigate the sensitivity of our results with respect to the length of our MD simulations. To this end, we recompute the rms inclination, θ_{rms}, and the plateau value of the normalised angle autocorrelation function, $\mathcal{C}_\theta(\tau \to \infty)$, for a set of shorter sampling times $t_{\text{sampling}} = \{100, 250, 500, 1000\}$ ps. We then compare these values with those reported in the manuscript for three different systems with varying defect concentrations. These systems encompass the pristine reference, the highest defect concentration of 1.0% (36 defects), and a sample at the critical defect concentration of $\approx 0.1\%$ (4 defects), where the phase transition occurs. The original computations for all of these systems were conducted over sampling times of approximately 1.5 ns. Similar to our size dependence analysis in the previous section, we exclusively focus here on divacancy defects but expect analogous trends for systems with Stone-Wales defects. A summary of our convergence analysis is shown in figure S20.

For both properties, the qualitative differences between the three systems remain apparent regardless of the simulation time.

The rms inclination shows hardly any dependence, with the deviation between lowest and longest sampling time being below 5% for all systems being. The plateau value of the normalised angle autocorrelation function, $\mathcal{C}_\theta(\tau \to \infty)$, conversely, shows a slightly stronger dependence for the graphene system at the critical defect concentration. Naturally, dynamical properties are inherently more challenging to converge which is also reflected in a higher uncertainty denoted by the larger errorbars. However, for all three systems both properties seem to have plateaued at a simulation length of 1 ns. Considering our shortest sampling time is 700 ps, and the majority of our simulations exceed 1 ns in duration, we can conclude that our results are sufficiently converged and allow for a quantitative comparison between the all systems.

As a final aspect of our time scale convergence analysis, we demonstrate that the chosen simulation times of approximately 1 ns are sufficient to fully capture the system dynamics, including those at the lowest relevant frequency. To this end, we compute the characteristic frequency of the out-of-plane fluctuations in pristine graphene using the autocorrelation function of the atomic heights, $\Phi_h(\tau)$, defined as:

$$\Phi_h(\tau) = \langle h(\tau)h(0)\rangle = \frac{1}{N_a N_f}\sum_i^{N_a}\sum_j^{N_f}(h_i(t_j+\tau)h_i(t_j)) \ , \qquad [10]$$

where $h_i(t_j)$ is the out-of-plane height of atom i at time t_j, and the brackets $\langle ... \rangle$ correspond to an ensemble average. Given the explicit relationship between average ripple amplitude and system size, we compute $\Phi_h(\tau)$ for graphene of both system sizes investigated in the previous section, namely 1800 and 7200 atoms. To facilitate this comparison, we normalise $\Phi_h(\tau)$ to obtain the normalised height autocorrelation function (HACF) $\mathcal{C}_h(\tau)$ expressed as:

$$\mathcal{C}_h(\tau) = \frac{\Phi_h(\tau)}{\Phi_h(0)}. \qquad [11]$$

The normalised HACF as a function of correlation time for pristine graphene of both system sizes is depicted in figure S21. For both cases, we observe damped periodic oscillations which to the graphene sheet moving down (values below 0) and up (values above 0). The frequency and decorrelation time of these oscillations exhibit a strong dependence on system size. For the smaller system comprising 1800 atoms, the normalized HACF reveals a frequency of approximately 100 GHz, corresponding to a period of about 10 ps, and asymptotically approaches zero for correlation times greater than 50 ps. In contrast, the larger system with 7200 atoms shows a lower frequency of approximately 30 GHz and a significantly longer decorrelation time exceeding 100 ps. The higher oscillation frequencies arise from the constraints imposed by the periodic boundary conditions, which limit the amplitude of the oscillations and consequently reduce the time the membrane requires to transition between its maximum out-of-plane displacements. In the previous section, we investigated the impact of system size on the results presented in the manuscript. Building on those findings, we now also conclude that the frequencies relevant to the system size investigated in the manuscript are adequately sampled within the chosen observation time of 1 ns.

S3.B. Alternative observables . The objective of this section is to validate the robustness of our results by comparing the measures established in the manuscript to alternative observables. To this end, we first compare our rms inclination to the corrugation amplification factor (CAF) introduced in reference (2). Subsequently, we will also compute the mean-squared displacement (MSD) in the direction perpendicular to the sheet and the velocity probability density function (PDF) which have been reported for pristine graphene in reference (17).

S3.B.1. Corrugation amplification factor. The corrugation amplification factor (CAF) aims to quantify the corrugation of a graphene sheet with respect to the pristine reference. Specifically, the CAF represents the ratio of the standard deviation of the atomic heights distribution sampled over all atoms and frames of a system relative to that observed for pristine graphene. For a more comprehensive explanation the reader is referred to the original reference (2). Here, we compute the CAF for all divacancy systems studied in the manuscript and an overview of the results and a comparison to the rms inclination is shown in figure S22.

Starting with the left panel of figure S22, we find that the CAF and the rms inclination exhibit a very similar dependence on the defect concentration. Specifically, the divacancy concentration with highest variance across different spatial arrangements is identical for both quantities. However, the CAF exhibits a significantly larger statistical error. This can be attributed mainly to two factors: First, the propagation of uncertainty inherently induced by relating all measurements to the value computed for pristine graphene. Second, by assuming the atomic heights follow a unimodal normal distribution which may not be accurate for highly defective systems. Note that compared to the original reference (2), here we use a more conservative error estimate by taking the three-fold standard deviation obtained from bootstrapping over 4 blocks rather than the single standard deviation derived from block averaging over 20 blocks. Despite these shortcomings, the CAF seems to qualitatively confirm the well-converged results based on the rms inclination. In fact, as shown in the left panel of figure S22, there is a strong correlation between the two quantities.

S3.B.2. Mean-squared displacement. Turning now to an alternative measurement to quantify the dynamics of the system, here we focus on the mean-squared displacement (MSD) of the graphene sheet perpendicular to the surface. Previous experiments on pristine graphene (17) observed two distinct regimes where superdiffusive and subdiffusive motion dominated at short and long times, respectively. Here, we compute the MSD over 200 ps for the same set of systems as in section S3.A.3, ranging from

pristine graphene to the highest divacancy concentration of 1.0% (36 defects). To understand the sensitivity of the MSD with respect to the arrangements of defects, we further analyse the MSD for all samples at the critical divacancy concentration of $\approx 0.1\%$ (4 defects). Due to the large time scale, here we compute the statistical error based on the standard deviation obtained via block averaging over 4 blocks rather than performing bootstrapping. A summary of the obtained results is depicted in figure S23 with both the MSD and the time represented on a logarithmic scale.

Similar to the experiments (17), we observe two distinct regimes for pristine graphene (*Pristine*): rapid diffusion at short time scales and slow motion at longer scales. For times ≥ 10 ps we observe damped periodic oscillations which can be attributed to the finite size of our simulation box and their frequency intrinsically dependent on the system size. The most defective system (*High*) shows overall a very similar behaviour compared to prisitine graphene, with the exception of a slower diffusion at shorter time scales and a shift towards lower MSDs in the long-term limit. This agrees well with our findings indicating a less mobile system at large defect concentrations. Most intriguing, however, is the behaviour of the system at the critical defect concentration (*Transition*): While it mimics the motion of pristine graphene at short time scales, the MSD converges to a larger value without showing the oscillations observed for the two other systems.

To better understand these findings, the left subplot of figure S23 displays the MSD as a function of time for all spatial realisations of the critical divacancy concentration studied in this work. While all systems seem to behave identically at short time scales, they vary significantly at times ≥ 10 ps. In particular, one of the systems, denoted as DV4 2, displays a comparable pattern to the highly defective reference system, characterised by oscillations and ultimately converging to a MSD lower than that of pristine graphene. Interestingly, DV4 2 exhibits the largest value of $\mathcal{C}_\theta(\tau \to \infty) \approx 0.9$ (cf. figure S3) at this given concentration. This suggests a direct relationship between the MSD, and thus, the mobility of the system, with the introduced plateau value of the angle autocorrelation function. Furthermore, the relatively larger MSDs in the long-time limit suggest that with increasing defect concentration, the system initially becomes more mobile, at least locally, before eventually converging to a highly static pattern when more defects are introduced. This aligns well with our findings regarding local areas of high mobility, as demonstrated in the atomic heatmaps of high concentrations of both divacancies and Stone-Wales defects analysed in the previous section and depicted in Figure 3 of the manuscript.

S3.B.3. Velocity probability distribution. Considering the experimental reports (17) suggesting that the out-of-plane velocity of pristine graphene follows a Cauchy-Lorentz distribution rather than a Normal distribution, we proceed to compute the velocities of the atoms in the direction perpendicular to the sheet using finite differences. We then compute the probability distribution over all atoms and frames where we employ bootstrapping with 4 blocks to estimate the statistical error. In an analogous fashion with the previous sections, this analysis is carried out for three different systems with varying divacancy concentration. These systems include the pristine reference, the highest defect concentration of 1.0% (36 divacancies), and a sample at the critical divacancy concentration of $\approx 0.1\%$ (4 defects). A summary of our results is displayed in figure S24. In contrast to the experiments, we find that all graphene membranes follow a Normal distribution irrespective of their defect concentration.

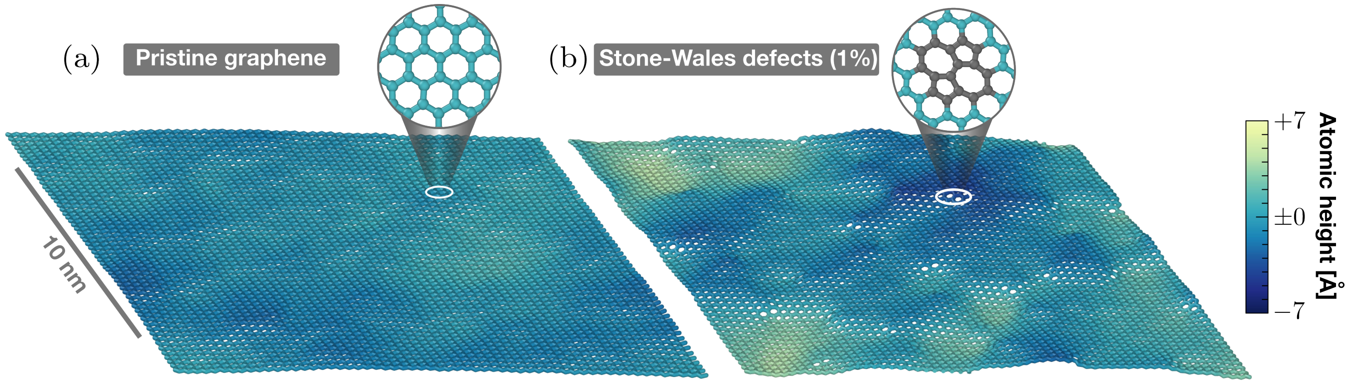

Fig. S1. Impact of Stone-Wales defects on the structure of free-standing graphene. The atoms in (a) pristine and (b) 1% Stone-Wales (inset) defective free-standing graphene are colored according to their out-of-plane position relative to the center of mass of the respective system. Same color code as Fig. 1 (main text). While the corrugation induced by Stone-Wales defects is smaller than divacancies, it is still larger than in pristine graphene. We also show in the main text that a large concentration of Stone-Wales defects affects the rippling dynamics of the sheet.

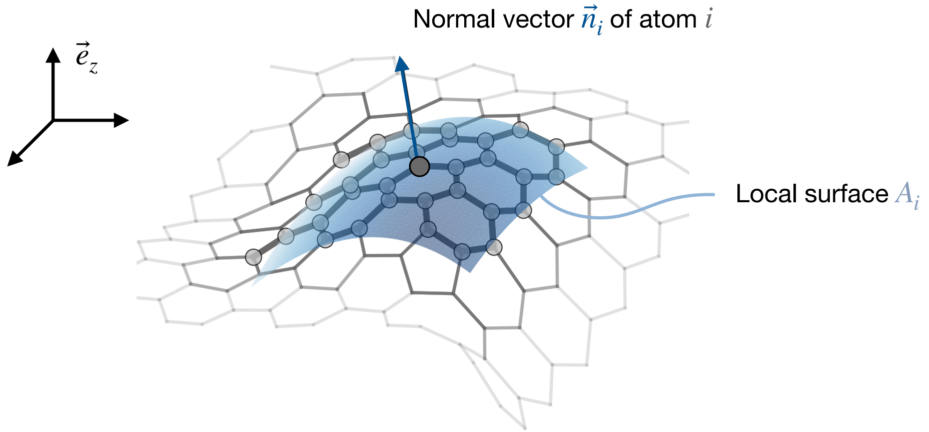

Fig. S2. Schematic illustration of the atomic normal vector, $\vec{n}_i$, and the local surface, A_i, for an arbitrary atom i in graphene. The atoms highlighted by the spheres are used to fit the coefficients of the polynomial to approximate A_i.

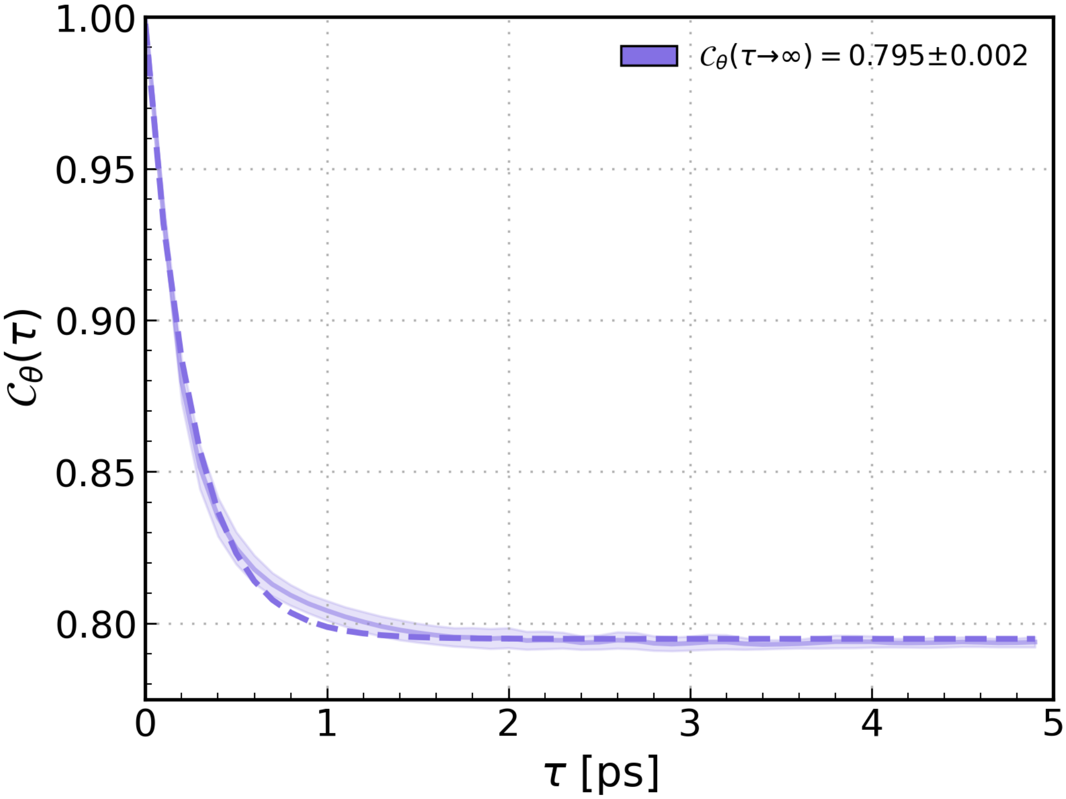

Fig. S3. Angle autocorrelation function, $\mathcal{C}_\theta(\tau)$, for pristine graphene. The continuous line corresponds to the values measured in our simulations, while the shaded area represents the threefold standard deviation estimated via bootstrapping. The dashed line corresponds to the fitted exponential function in equation 8 and in the legend we report the estimated plateau value based on this fit.

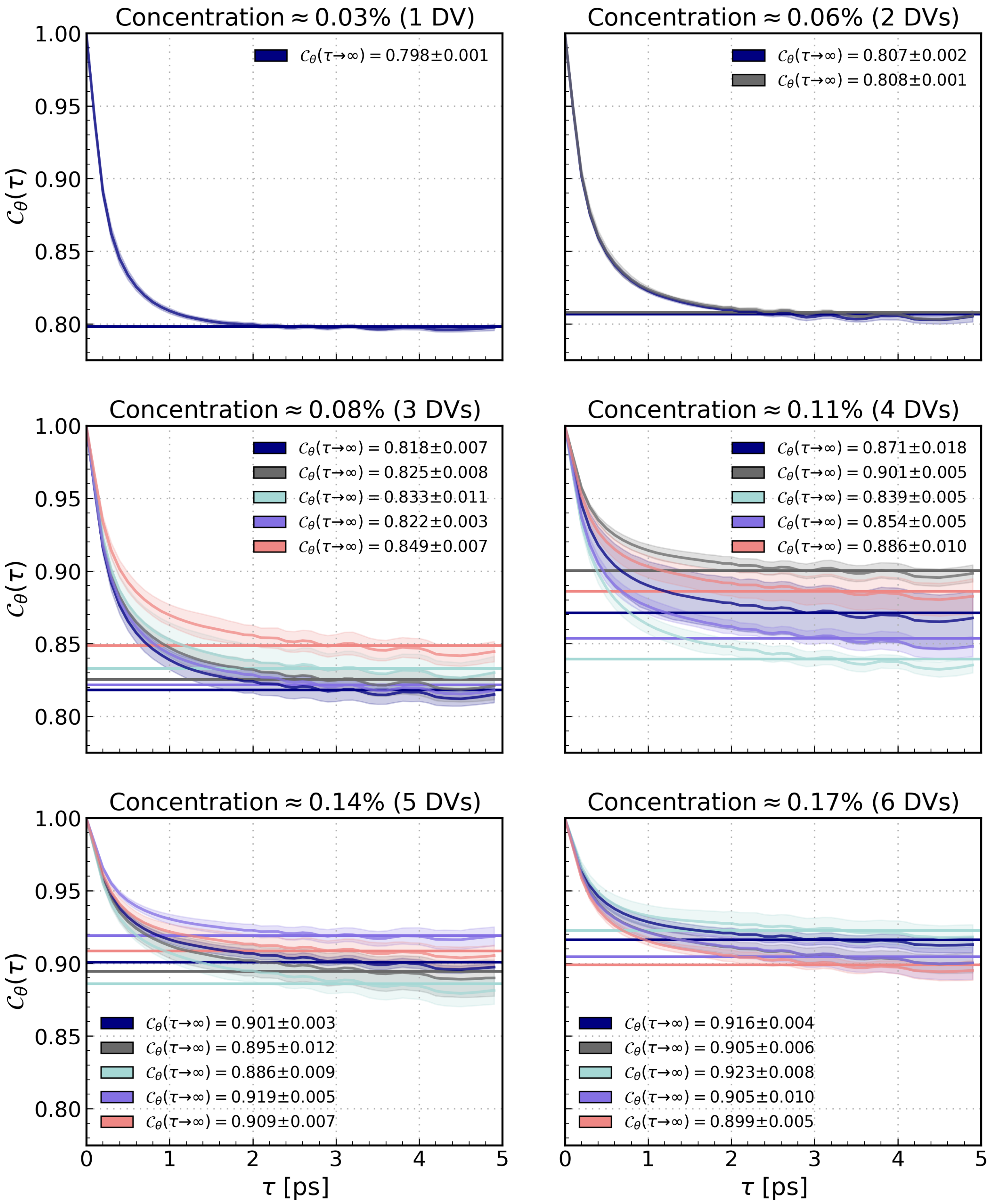

Fig. S4. Angle autocorrelation function, $\mathcal{C}_\theta(\tau)$, for systems comprising 1 to 6 divacancy defects corresponding to defect concentrations between $\approx 0.03\%$ and $\approx 0.17\%$. Each subplot illustrates between 1 and 5 systems of identical defect concentration but arranged in various spatial arrangements. The continuous line corresponds to the values measured in our simulations, while the shaded area represents the threefold standard deviation estimated via bootstrapping. The plateau value approximated by the constant c of the exponential function is illustrated by the horizontal line. For each system, the corresponding plateau values with their associated uncertainty are given in the legend.

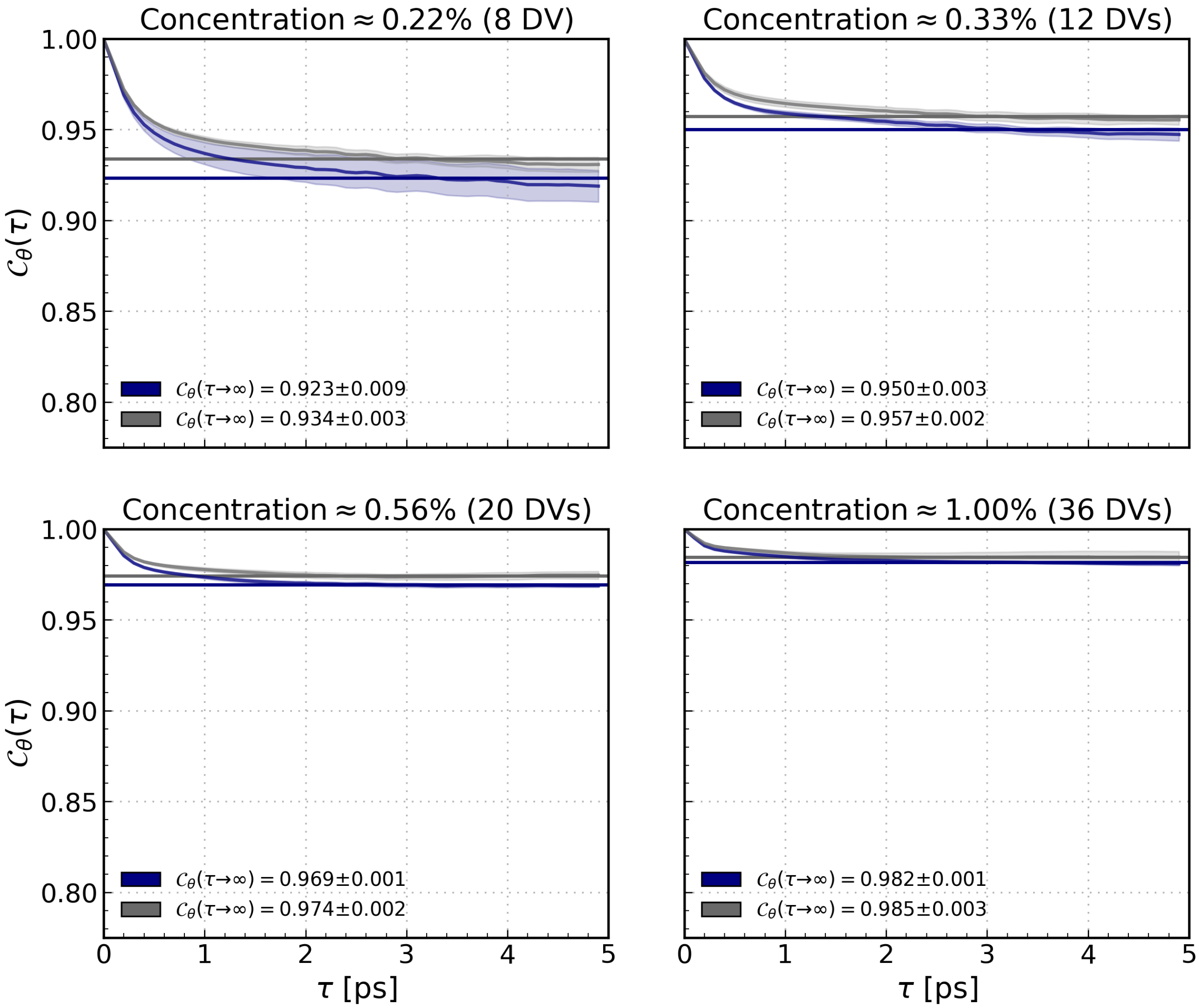

Fig. S5. Angle autocorrelation function, $\mathcal{C}_\theta(\tau)$, for systems comprising 8 to 36 divacancy defects corresponding to defect concentrations between $\approx 0.22\%$ and 1.00%. Each subplot illustrates 2 systems of identical defect concentration but arranged in various spatial arrangements. The continuous line corresponds to the values measured in our simulations, while the shaded area represents the threefold standard deviation estimated via bootstrapping. The plateau value approximated by the constant c of the exponential function is illustrated by the horizontal line. For each system, the corresponding plateau values with their associated uncertainty are given in the legend.

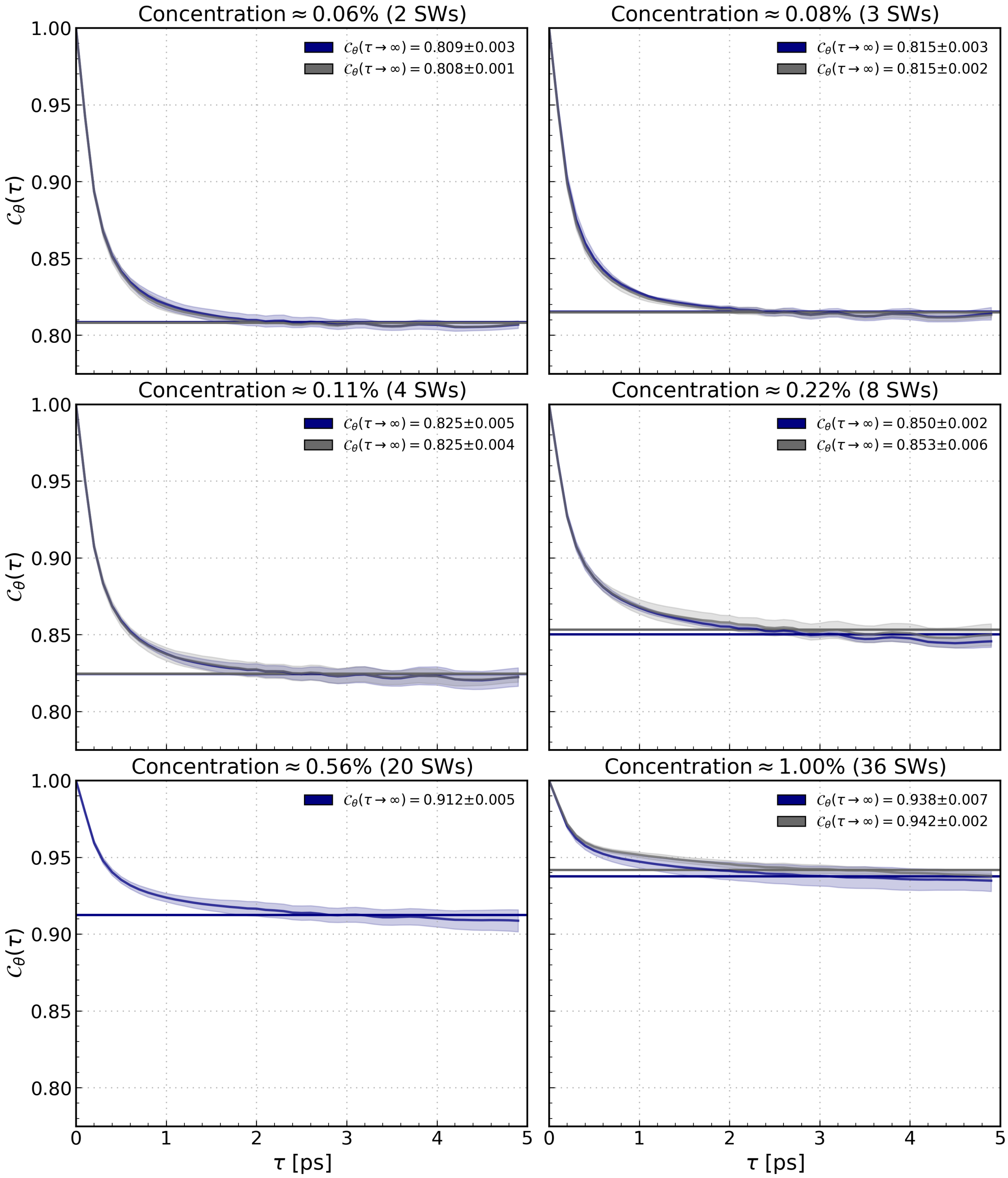

Fig. S6. Angle autocorrelation function, $\mathcal{C}_\theta(\tau)$, for systems comprising 2 to 36 Stone-Wales defects corresponding to defect concentrations between $\approx 0.06\%$ and 1.00%. Each subplot illustrates 2 systems of identical defect concentration but arranged in various spatial arrangements. The continuous line corresponds to the values measured in our simulations, while the shaded area represents the threefold standard deviation estimated via bootstrapping. The plateau value approximated by the constant c of the exponential function is illustrated by the horizontal line. For each system, the corresponding plateau values with their associated uncertainty are given in the legend.

Table S1. Accuracy and convergence metrics related to the calculation of an atom-resolved $\mathcal{C}_\theta(\tau \to \infty)$ for each system.

System	Concentration [%]	Lowest R^2 score	#Atoms with $R^2 < 0.8$
Pristine	0.000	0.971	0
DV1 1	≈ 0.028	0.955	0
DV2 1	≈ 0.056	0.945	0
DV2 2		0.946	0
DV3 1	≈ 0.083	0.934	0
DV3 2		0.924	0
DV3 3		0.917	0
DV3 4		0.921	0
DV3 5		0.867	0
DV4 1	≈ 0.111	0.809	0
DV4 2		0.803	0
DV4 3		0.892	0
DV4 4		0.844	0
DV4 5		0.835	0
DV5 1	≈ 0.139	0.650	48
DV5 2		0.803	0
DV5 3		0.792	2
DV5 4		0.706	30
DV5 5		0.763	10
DV6 1	≈ 0.167	0.754	5
DV6 2		0.808	0
DV6 3		0.808	0
DV6 4		0.770	7
DV6 5		0.738	16
DV8 1	≈ 0.222	0.700	9
DV8 2		0.771	5
DV12 1	≈ 0.333	0.714	62
DV12 2		0.728	31
DV20 1	≈ 0.556	0.537	173
DV20 2		0.624	159
DV36 1	1.000	0.562	528
DV36 2		0.525	785

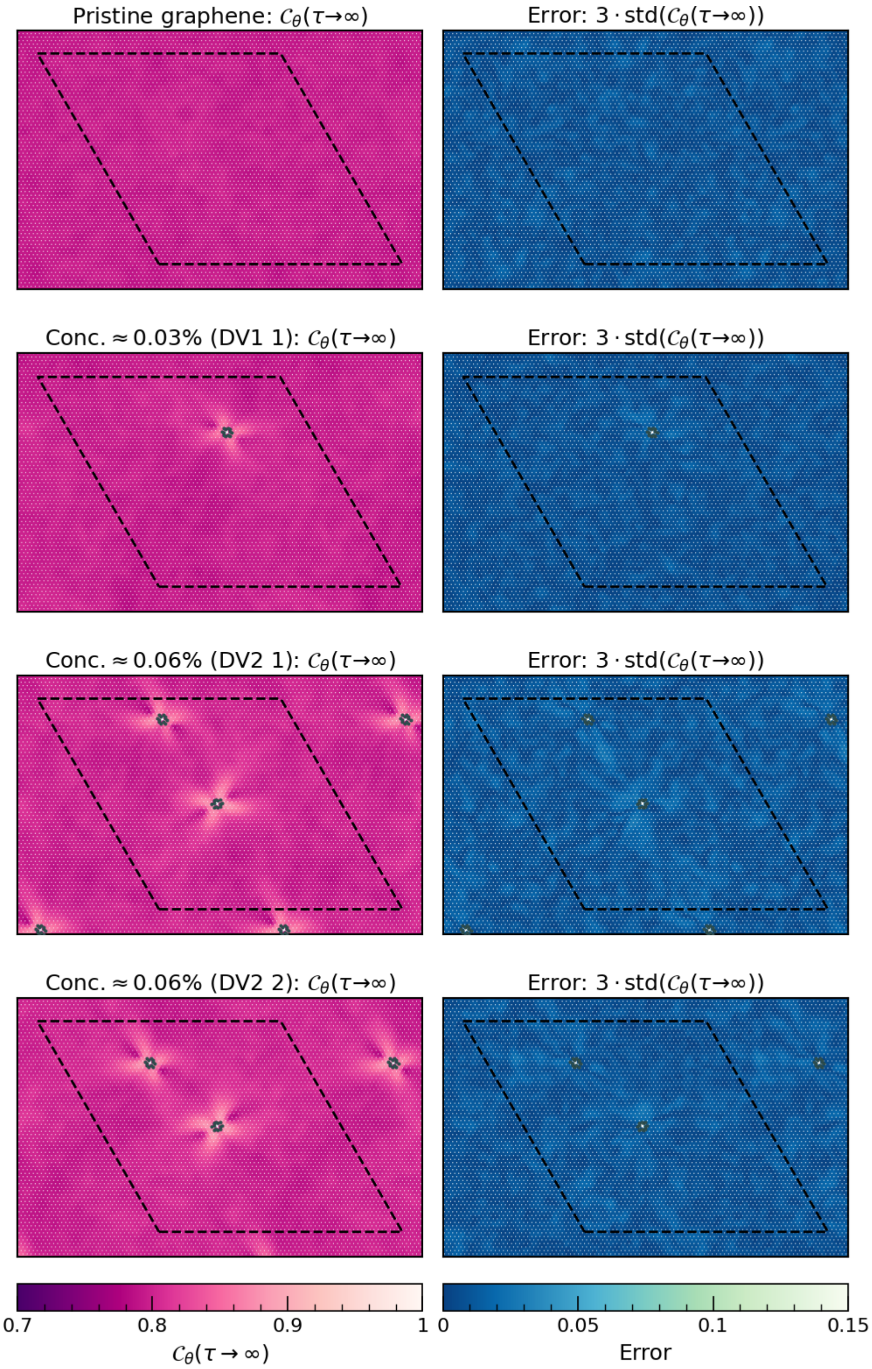

Fig. S7. Atom-resolved plateau values of the angle autocorrelation function, $\mathcal{C}_\theta(\tau\to\infty)$, for systems comprising up to 2 divacancy defects corresponding to a defect concentration of up to $\approx 0.06\%$. The atoms forming a divacancy are highlighted in grey. For each system we report the calculated atomic values (left) as well as the associated error based on the threefold standard deviation estimated via bootstrapping (right).

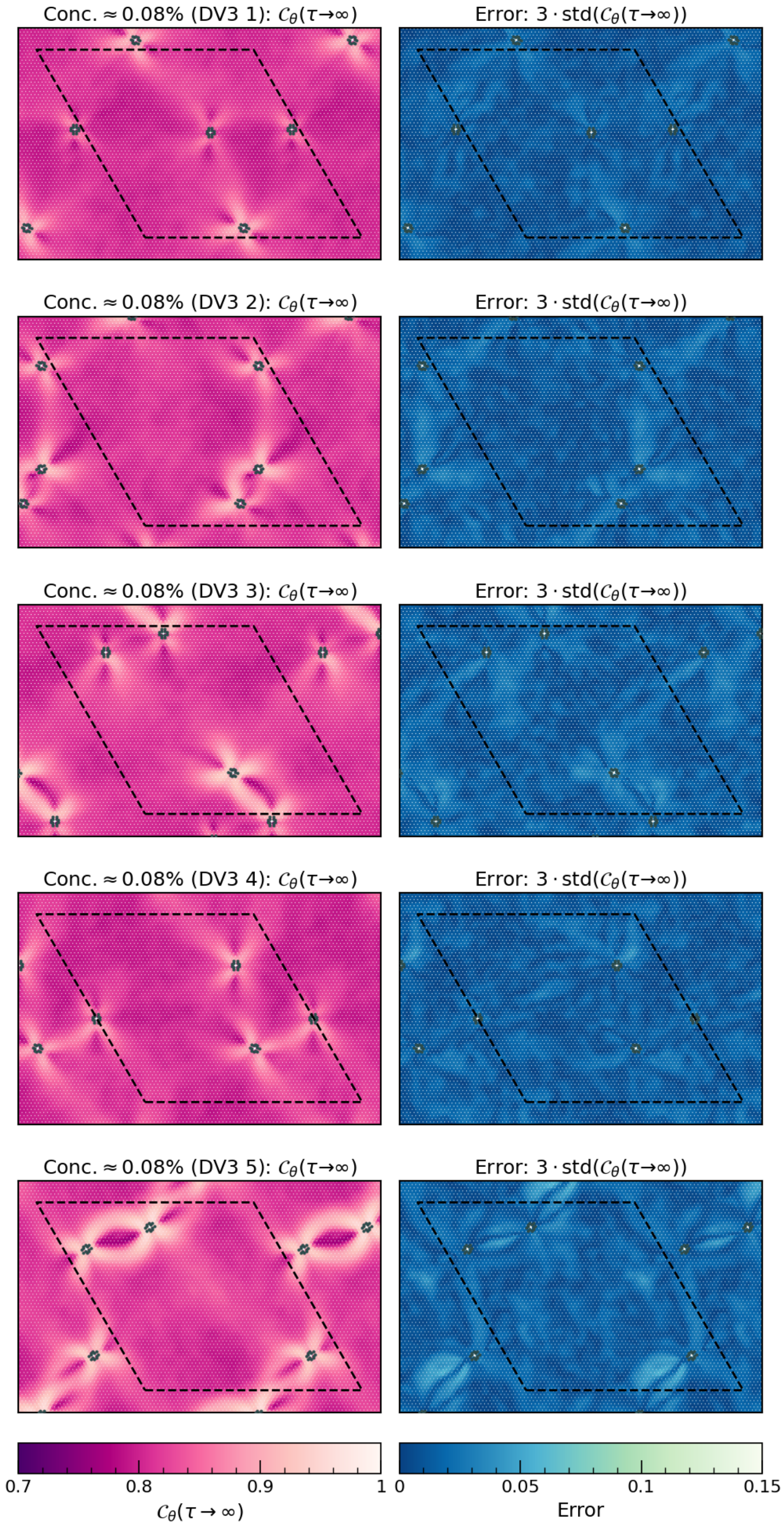

Fig. S8. Atom-resolved plateau values of the angle autocorrelation function, $\mathcal{C}_\theta(\tau \to \infty)$, for systems comprising 3 divacancy defects corresponding to a defect concentration of $\approx 0.08\%$. The atoms forming a divacancy are highlighted in grey. For each system we report the calculated atomic values (left) as well as the associated error based on the threefold standard deviation estimated via bootstrapping (right).

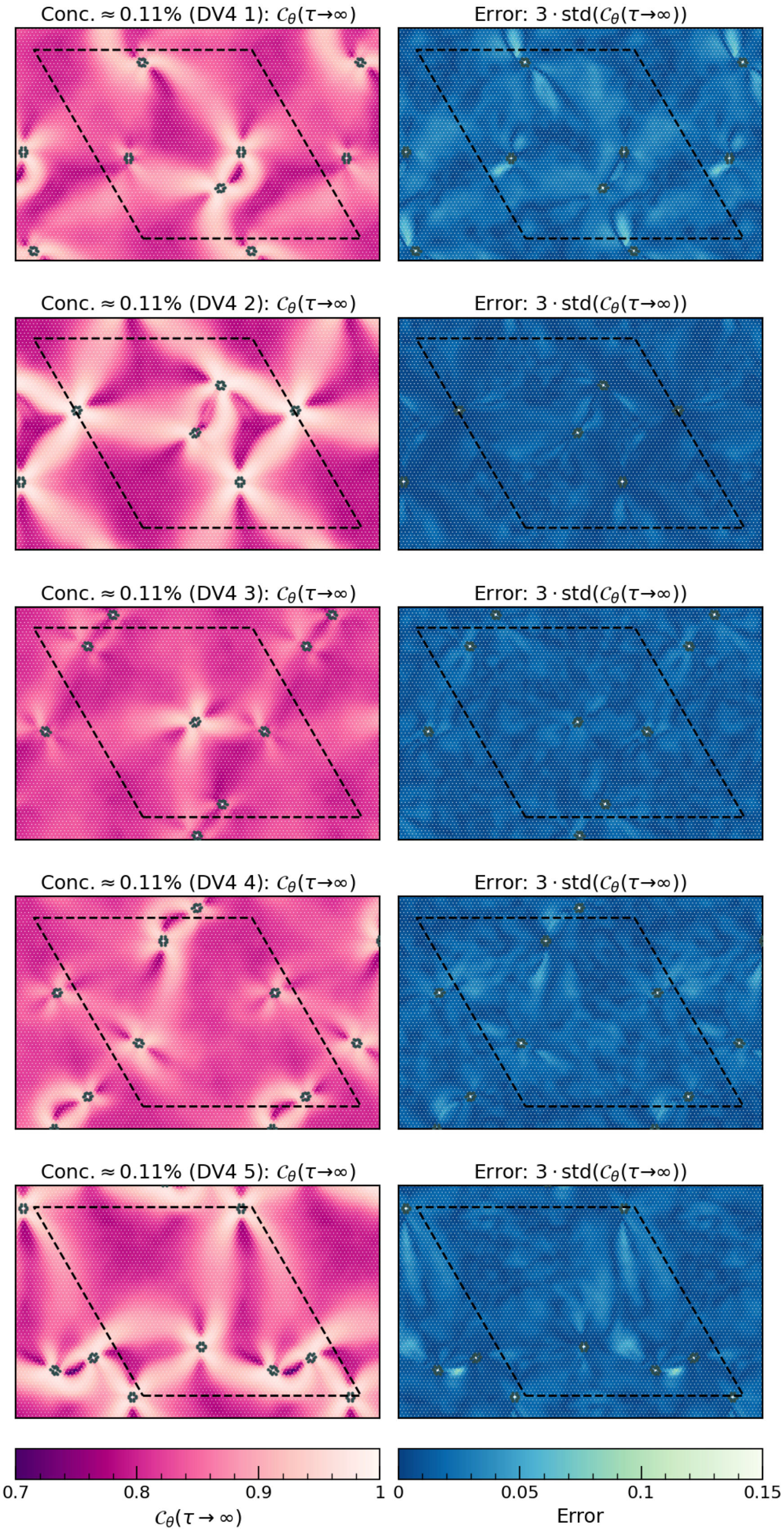

Fig. S9. Atom-resolved plateau values of the angle autocorrelation function, $\mathcal{C}_\theta(\tau \to \infty)$, for systems comprising 4 divacancy defects corresponding to a defect concentration of $\approx 0.11\%$. The atoms forming a divacancy are highlighted in grey. For each system we report the calculated atomic values (left) as well as the associated error based on the threefold standard deviation estimated via bootstrapping (right).

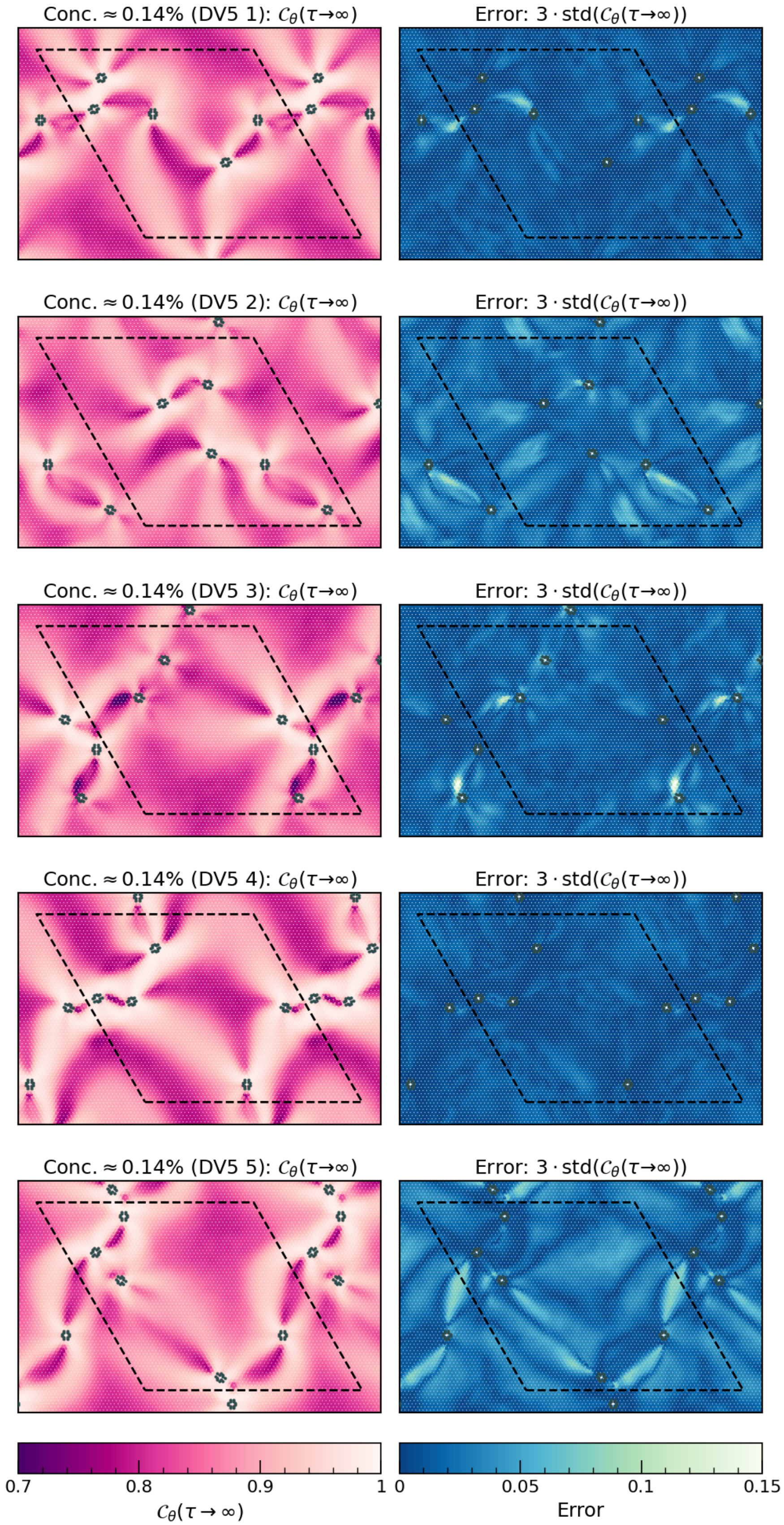

Fig. S10. Atom-resolved plateau values of the angle autocorrelation function, $\mathcal{C}_\theta(\tau\to\infty)$, for systems comprising 5 divacancy defects corresponding to a defect concentration of $\approx 0.14\%$. The atoms forming a divacancy are highlighted in grey. For each system we report the calculated atomic values (left) as well as the associated error based on the threefold standard deviation estimated via bootstrapping (right).

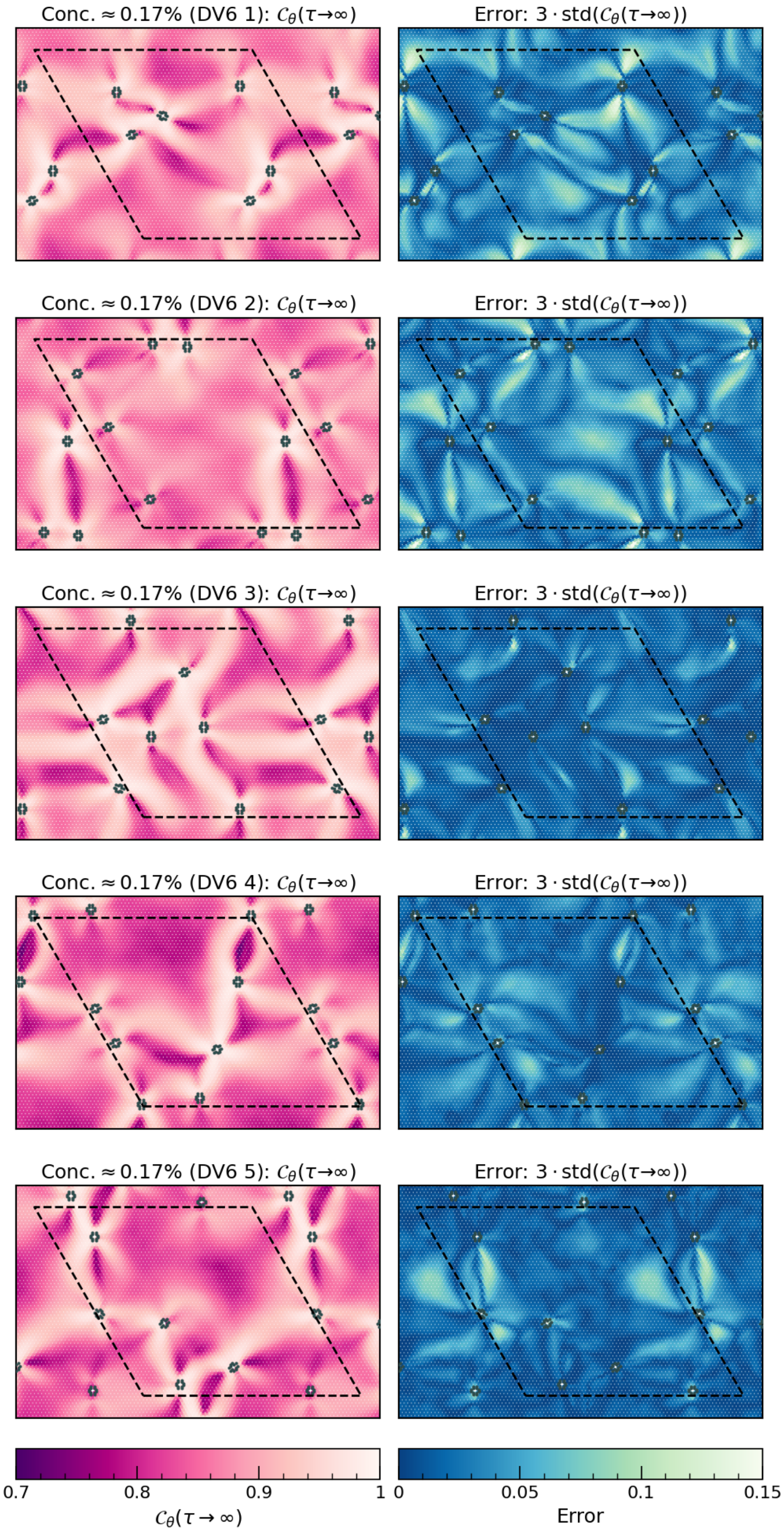

Fig. S11. Atom-resolved plateau values of the angle autocorrelation function, $\mathcal{C}_\theta(\tau \to \infty)$, for systems comprising 6 divacancy defects corresponding to a defect concentration of $\approx 0.17\%$. The atoms forming a divacancy are highlighted in grey. For each system we report the calculated atomic values (left) as well as the associated error based on the threefold standard deviation estimated via bootstrapping (right).

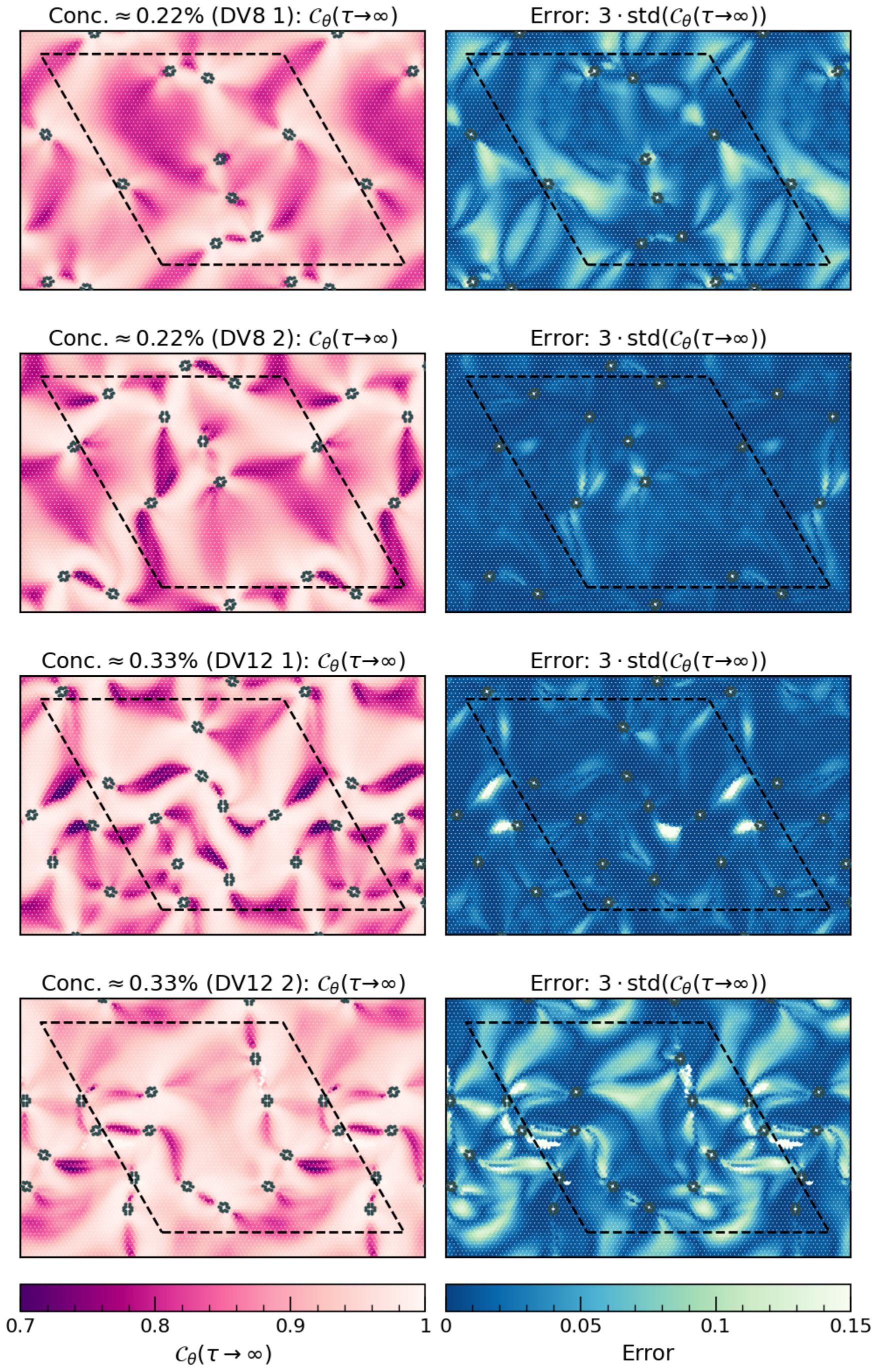

Fig. S12. Atom-resolved plateau values of the angle autocorrelation function, $\mathcal{C}_\theta(\tau \to \infty)$, for systems comprising between 8 and 12 divacancy defects corresponding to a defect concentration between $\approx 0.22\%$ and $\approx 0.33\%$. The atoms forming a divacancy are highlighted in grey. For each system we report the calculated atomic values (left) as well as the associated error based on the threefold standard deviation estimated via bootstrapping (right). White areas correspond to atoms where the prediction or error estimate are outside the color scale and are, thus, not reliable results.

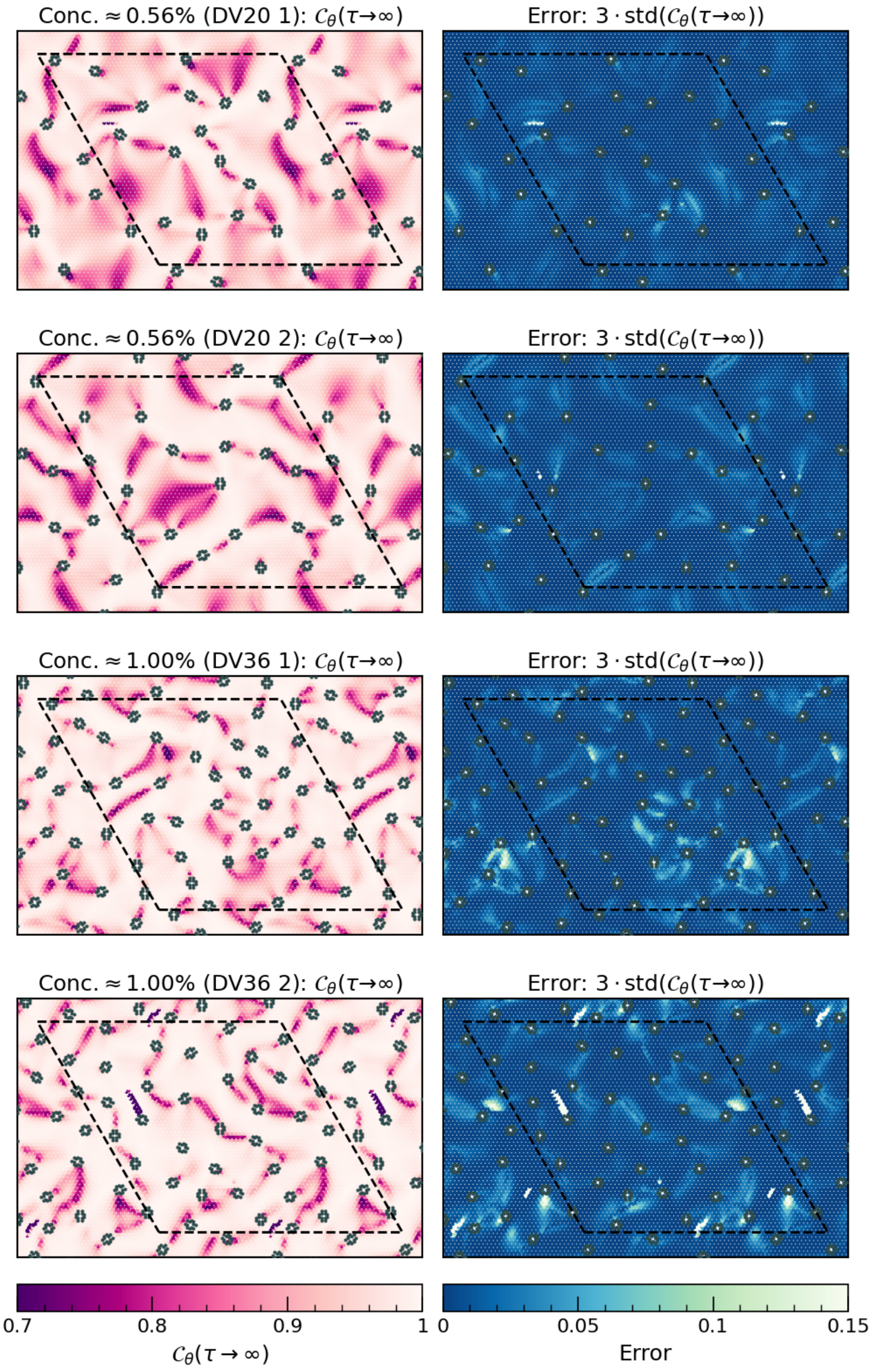

Fig. S13. Atom-resolved plateau values of the angle autocorrelation function, $\mathcal{C}_\theta(\tau \to \infty)$, for systems comprising between 20 and 36 divacancy defects corresponding to a defect concentration between $\approx 0.56\%$ and 1%. The atoms forming a divacancy are highlighted in grey. For each system we report the calculated atomic values (left) as well as the associated error based on the threefold standard deviation estimated via bootstrapping (right). White areas correspond to atoms where the prediction or error estimate are outside the color scale and are, thus, not reliable results.

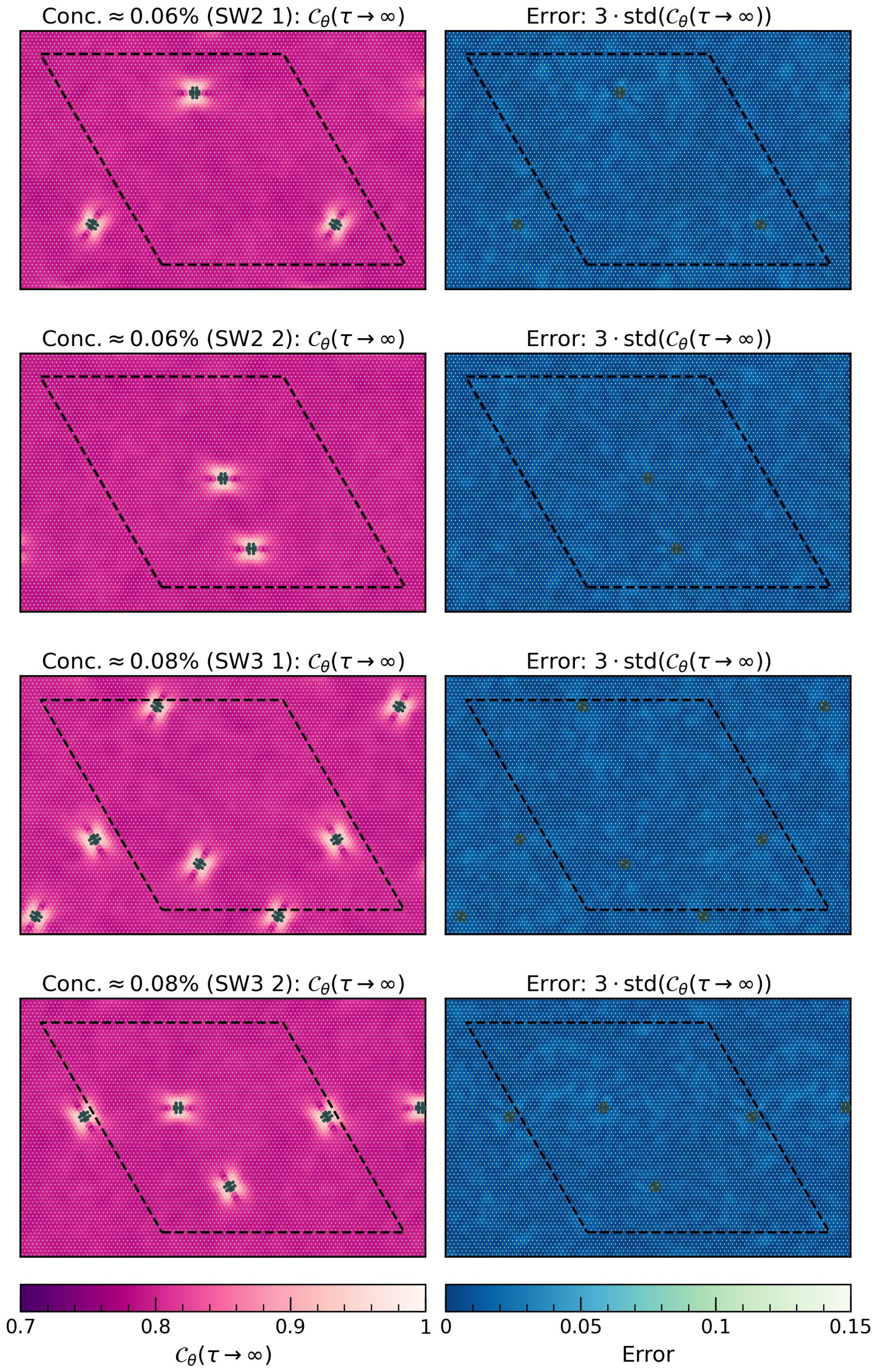

Fig. S14. Atom-resolved plateau values of the angle autocorrelation function, $\mathcal{C}_\theta(\tau \to \infty)$, for systems comprising 2 or 3 Stone-Wales defects corresponding to a defect concentration between $\approx 0.06\%$ and $\approx 0.08\%$. The atoms forming a Stone-Wales are highlighted in grey. For each system we report the calculated atomic values (left) as well as the associated error based on the threefold standard deviation estimated via bootstrapping (right). White areas correspond to atoms where the prediction or error estimate are outside the color scale and are, thus, not reliable results.

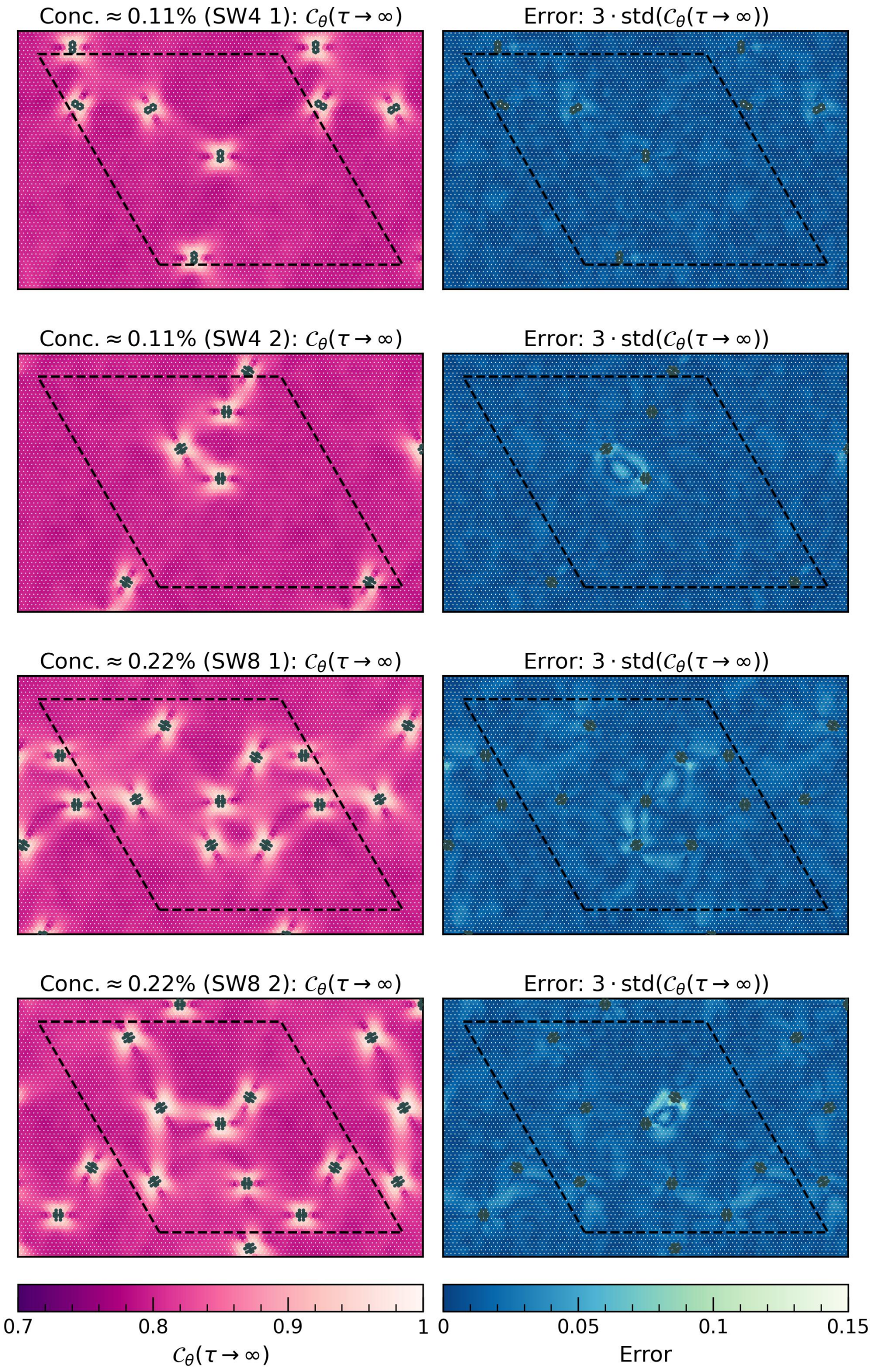

Fig. S15. Atom-resolved plateau values of the angle autocorrelation function, $\mathcal{C}_\theta(\tau \rightarrow \infty)$, for systems comprising 4 or 8 Stone-Wales defects corresponding to a defect concentration between $\approx 0.11\%$ and $\approx 0.22\%$. The atoms forming a Stone-Wales are highlighted in grey. For each system we report the calculated atomic values (left) as well as the associated error based on the threefold standard deviation estimated via bootstrapping (right). White areas correspond to atoms where the prediction or error estimate are outside the color scale and are, thus, not reliable results.

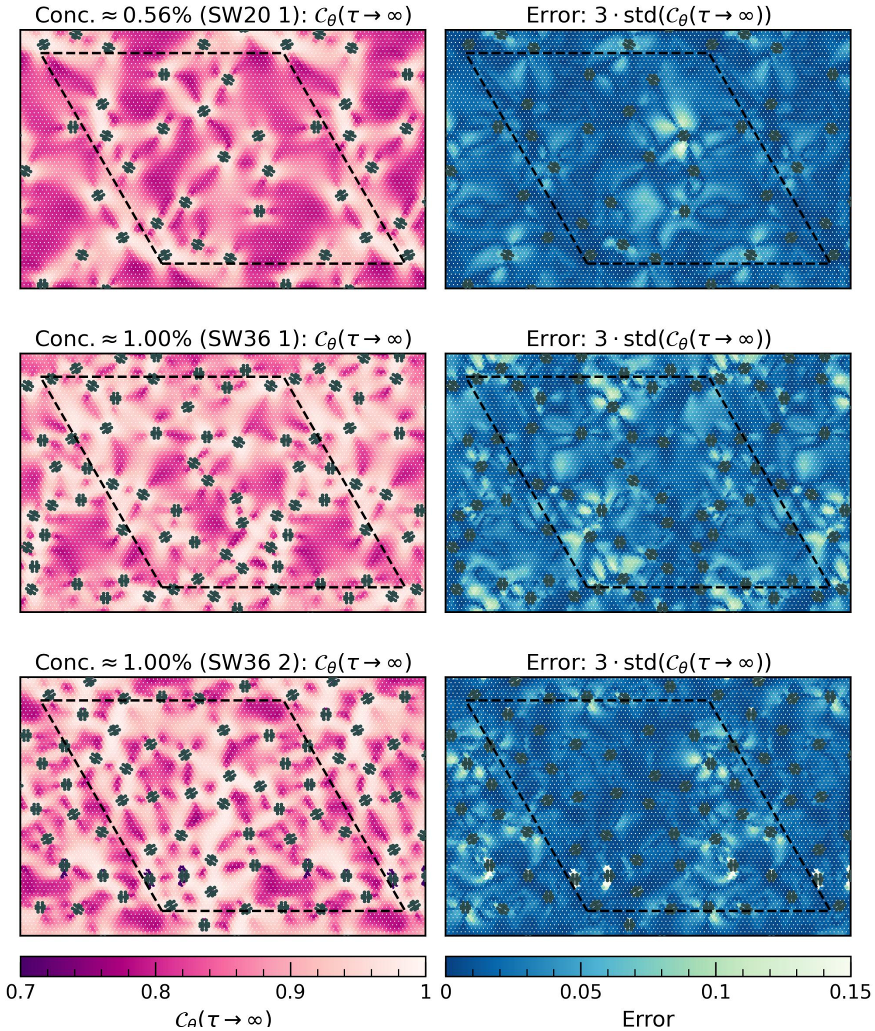

Fig. S16. Atom-resolved plateau values of the angle autocorrelation function, $\mathcal{C}_\theta(\tau \to \infty)$, for systems comprising 4 or 8 Stone-Wales defects corresponding to a defect concentration between $\approx 0.56\%$ and 1%. The atoms forming a Stone-Wales are highlighted in grey. For each system we report the calculated atomic values (left) as well as the associated error based on the threefold standard deviation estimated via bootstrapping (right). White areas correspond to atoms where the prediction or error estimate are outside the color scale and are, thus, not reliable results.

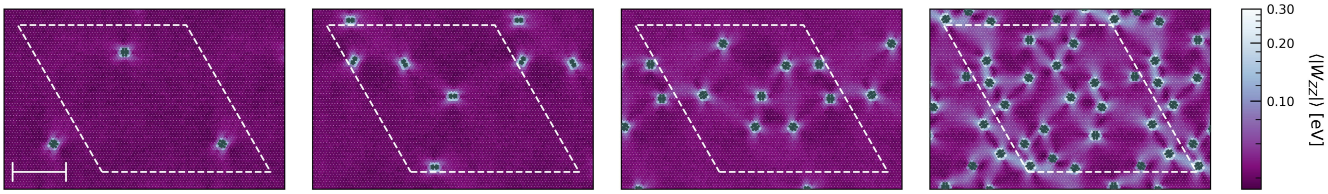

Fig. S17. Atom-resolved virial contribution to the stress along the surface normal for the systems comprising between 2 and 20 Stone-Wales defects corresponding to a defect concentration between $\approx 0.06\%$ and $\approx 0.56\%$. The atoms forming a Stone-Wales defect are highlighted in grey. The simulation box is indicated with dashed lines, and the scale bar drawn in the left subpanel represents 5 nm. The observed patterns and pathways are similar to those observed in the same systems when analyzing the atomic plateau values of the angle autocorrelation function, $\mathcal{C}_\theta(\tau \to \infty)$, as shown in figure 3 in the manuscript.

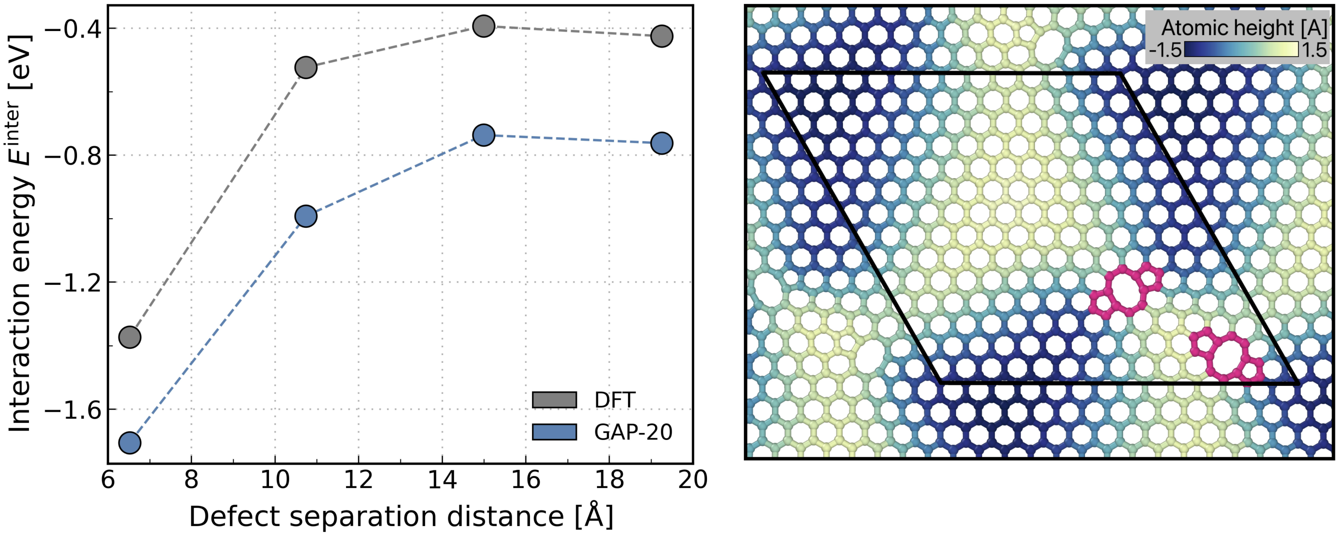

Fig. S18. Comparison of the defect interaction energy between the GAP-20 and DFT. The left panel depicts the defect interaction energy, E^{inter}, between two divacancy defects for four different defect separations, namely 6.52, 10.74, 14.99, and 19.25Å. The orientation between the two divacancies is identical across all systems. The dashed lines serve as guide to the eye. On the left panel, we show the minimum energy configuration predicted by the GAP-20 for a defect separation of 10.74Å. The atoms are coloured based on their atomic height and the particles forming the divacancies are highlighted in pink. The simulation box is shown by black lines.

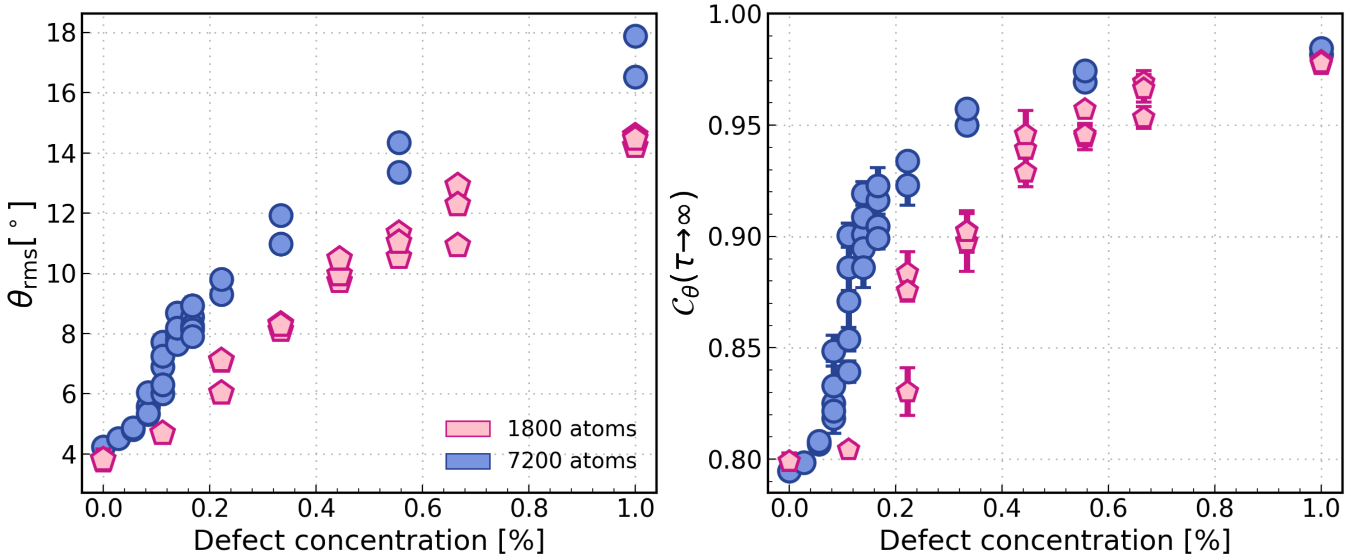

Fig. S19. Impact of system size on static and dynamic behaviour of graphene in the absence and presence of divacancy defects. The two panels show the rms inclination (left) and the plateau value of normalised angle autocorrelation function (right) as function of the defect concentration for two system sizes with the pristine reference systems containing 1800 and 7200 atoms, respectively. The data computed for the larger systems is identical to the pristine and divacancy data reported in the manuscript in Fig 2(a). The statistical error was computed for each system based on bootstrapping using four blocks and is visualised by the error bars to the threefold standard deviation.

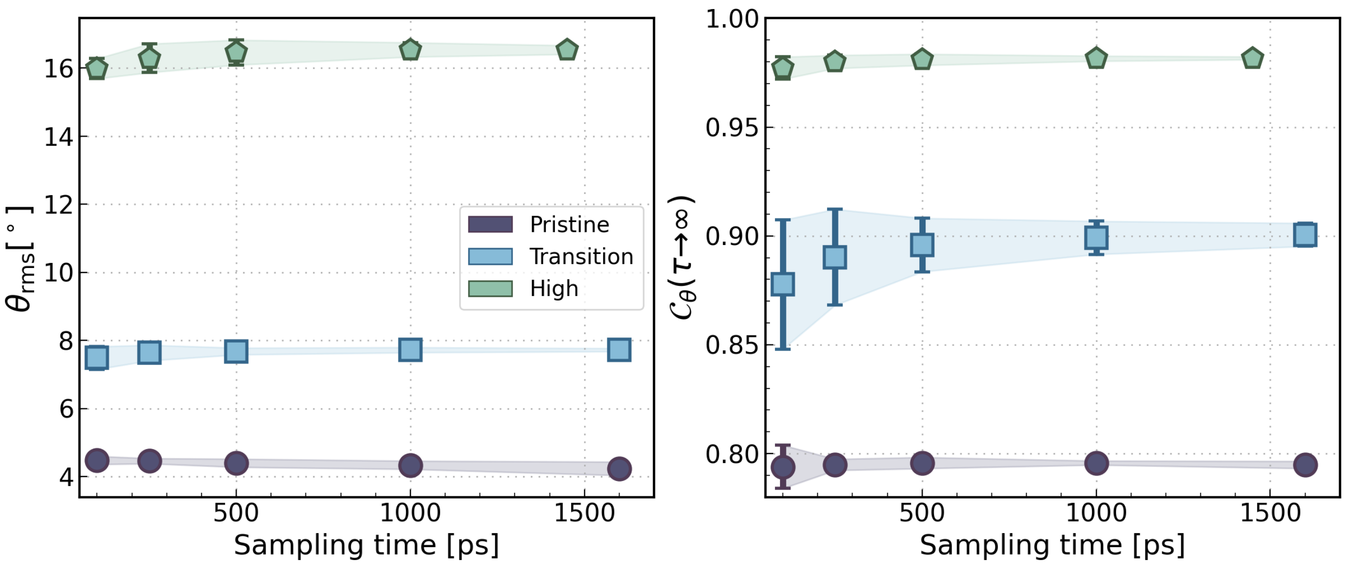

Fig. S20. Analysis of the convergence of static and dynamic behaviour of graphene in the absence and presence of divacancy defects with respect to the simulation time. The two panels show the rms inclination (left) and the plateau value of normalised angle autocorrelation function (right) against the sampling time for three distinct systems with varying defect concentration. In addition to a pristine sheet (*Pristine*), we analyse the convergence for graphene samples at the critical divacancy concentration of $\approx 0.1\%$ corresponding to 4 defects (*Transition*) and the most defective sample with 36 defects corresponding to a concentration of 1.0% (*High*). Values for the longest sampling times (around 1.5 ns) correspond to Fig 2(a) in the manuscript. Statistical errors, computed via bootstrapping using four blocks, are depicted with error bars, reflecting the threefold standard deviation, along with shaded areas.

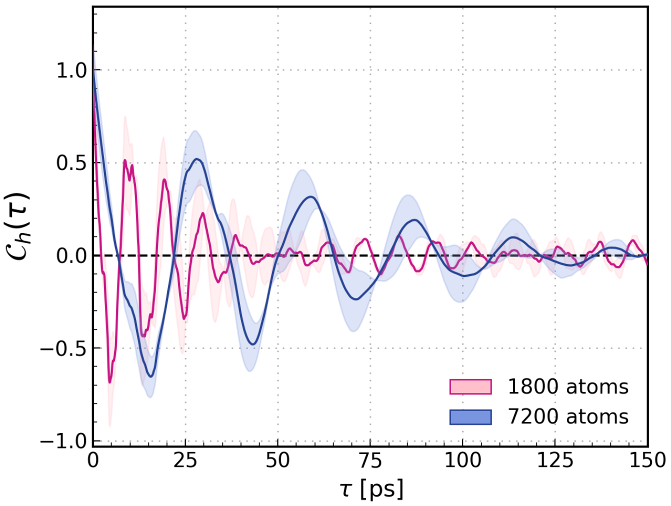

Fig. S21. Normalised height autocorrelation function (HACF) for pristine graphene comprising 1800 and 7200 atoms, respectively. The solid lines represent the actual data while the black dashed line is intended as a guide to the eye. The statistical error for each simulation is based on block averages using 2 blocks and visualised by the shading.

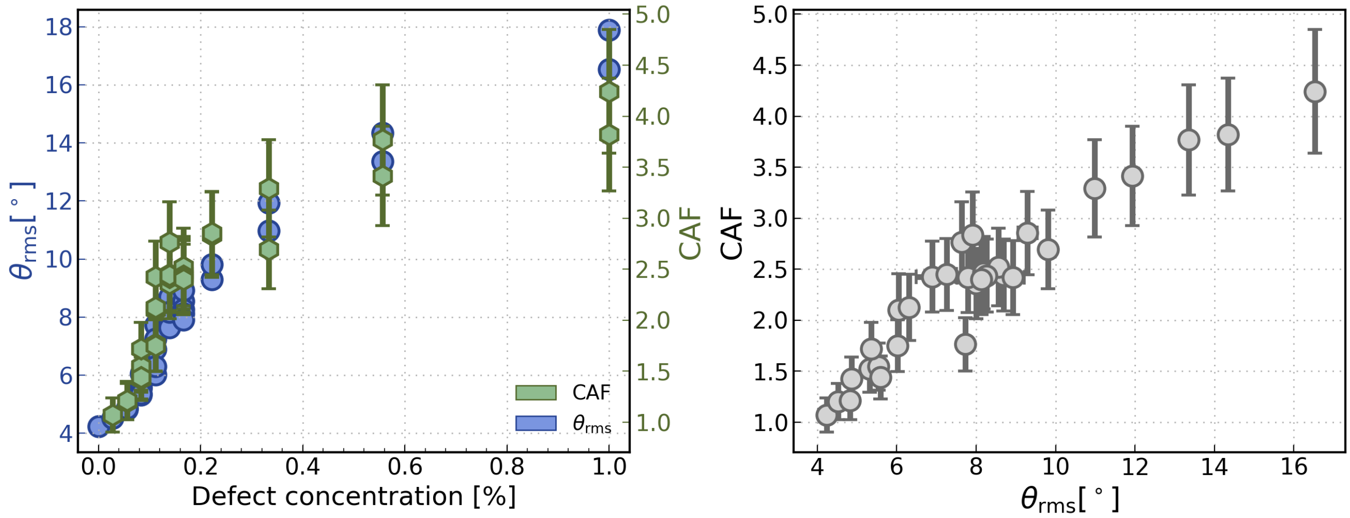

Fig. S22. Comparison between the rms inclination and the corrugation amplification factor (CAF) introduced in reference (2). The left panel shows both the CAF and the rms inclination as a function of the defect concentration. Note that the CAF for pristine graphene is 1 and not shown here. The data reported for the rms inclination is identical to that reported in the manuscript in figure 2a. To better understand the relationship between the two measures, the right panel displays the correlation between the two quantities. For both properties, the statistical errors were computed via bootstrapping using four blocks and are depicted with error bars, reflecting the threefold standard deviation.

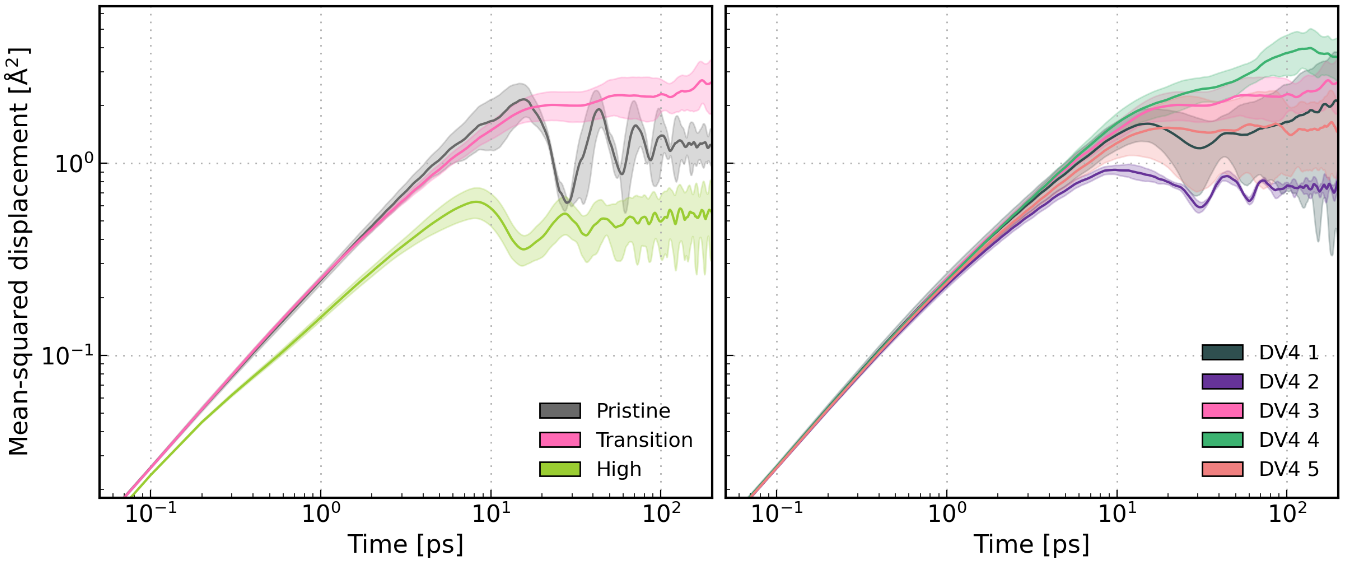

Fig. S23. Mean-squared displacement (MSD) of the graphene sheet height as a function of time. The left panel shows the MSD for three distinct systems with varying divacancy concentration. In addition to a pristine sheet (*Pristine*), we analyse the convergence for graphene samples at the critical divacancy concentration of $\approx 0.1\%$ corresponding to 4 divacancy defects (*Transition*) and the most defective sample with 36 divacancy defects corresponding to a concentration of 1.0% (*High*). The right subplot, conversely, displays the MSD for different spatial realisations of the critical defect concentration. The shaded areas represent the statistical error corresponding the standard deviation estimated via block averaging over 4 blocks.

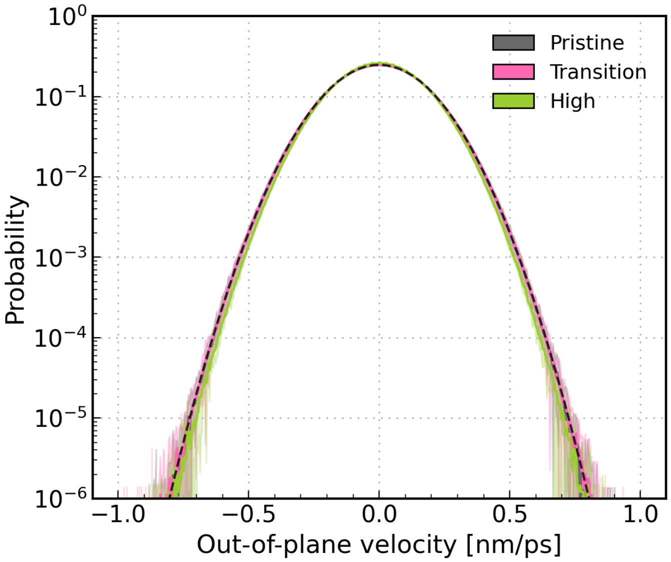

Fig. S24. Probability distribution of the atomic velocities perpendicular to the graphene sheet. In addition to a pristine sheet (*Pristine*), we analyse the convergence for graphene samples at the critical divacancy concentration of $\approx 0.1\%$ corresponding to 4 defects (*Transition*) and the most defective sample with 36 divacancy defects corresponding to a concentration of 1.0% (*High*). The dashed black line corresponds to a Gaussian fitted to the data of the *Pristine* system. The statistical errors were computed via bootstrapping using four blocks and are depicted with the shaded areas, reflecting the threefold standard deviation.

S4. Supplementary movies

Movie S1. Movie illustrating the dynamics of a pristine graphene sheet (Fig. 1a) over 1 ns.

Movie S2. Movie illustrating the dynamics of a defective graphene sheet with 0.055% defects (Fig. 3a) over 1 ns.

Movie S3. Movie illustrating the dynamics of a defective graphene sheet with 0.11% defects (Fig. 3b) over 1 ns.

Movie S4. Movie illustrating the dynamics of a defective graphene sheet with 0.11% defects (Fig. 3c) over 1 ns.

Movie S5. Movie illustrating the dynamics of a defective graphene sheet with 0.22% defects (Fig. 3d) over 1 ns.

References

1. P Rowe, VL Deringer, P Gasparotto, G Csányi, A Michaelides, An accurate and transferable machine learning potential for carbon. *J. Chem. Phys.* **153**, 034702 (2020).
2. FL Thiemann, P Rowe, A Zen, EA Müller, A Michaelides, Defect-Dependent Corrugation in Graphene. *Nano Lett.* **21**, 8143–8150 (2021).
3. S Plimpton, Fast Parallel Algorithms for Short-Range Molecular Dynamics. *J. Comput. Phys.* **117**, 1–19 (1995).
4. U Ludacka, et al., In situ control of graphene ripples and strain in the electron microscope. *npj 2D Mater. Appl.* **2**, 25 (2018).
5. G Kresse, J Hafner, Ab initio molecular dynamics for liquid metals. *Phys. Rev. B* **47**, 558–561 (1993).
6. G Kresse, J Furthmüller, Efficiency of ab-initio total energy calculations for metals and semiconductors using a plane-wave basis set. *Comput. Mater. Sci.* **6**, 15–50 (1996).
7. G Kresse, J Furthmüller, Efficient iterative schemes for ab initio total-energy calculations using a plane-wave basis set. *Phys. Rev. B* **54**, 11169–11186 (1996).
8. J Klimeš, DR Bowler, A Michaelides, Chemical accuracy for the van der waals density functional. *J. Physics: Condens. Matter* **22**, 022201 (2009).
9. JM Leyssale, GL Vignoles, A large-scale molecular dynamics study of the divacancy defect in graphene. *J. Phys. Chem. C* **118**, 8200–8216 (2014).
10. J Kotakoski, FR Eder, JC Meyer, Atomic structure and energetics of large vacancies in graphene. *Phys. Rev. B - Condens. Matter Mater. Phys.* **89**, 201406(R) (2014).
11. DR Nelson, L Peliti, Fluctuations in Membranes With Crystalline and Hexatic Order. *J. de physique Paris* **48**, 1085–1092 (1987).
12. P Le Doussal, L Radzihovsky, Self-consistent theory of polymerized membranes. *Phys. Rev. Lett.* **69**, 1209–1212 (1992).
13. DR Nelson, T Piran, S Weinberg, eds., *Statistical Mechanics of Membranes and Surfaces.* (World Scientific, Singapore), 2 edition, (2004).
14. JC Meyer, et al., The structure of suspended graphene sheets. *Nature* **446**, 60–63 (2007).
15. AK Singh, RG Hennig, Scaling relation for thermal ripples in single and multilayer graphene. *Phys. Rev. B - Condens. Matter Mater. Phys.* **87**, 094112 (2013).
16. R Singh, D Scheinecker, U Ludacka, J Kotakoski, Corrugations in free-standing graphene. *Nanomaterials* **12** (2022).
17. ML Ackerman, et al., Anomalous dynamical behavior of freestanding graphene membranes. *Phys. Rev. Lett.* **117**, 126801 (2016).
